# MicroRNA 29a therapy for CEACAM6-expressing lung adenocarcinoma

**DOI:** 10.1186/s12885-023-11352-w

**Published:** 2023-09-08

**Authors:** Seung-Myoung Son, Jieun Yun, Dong-Wook Kim, Young-Suk Jung, Sang-Bae Han, Yong Hee Lee, Hye Sook Han, Chang Gok Woo, Ho-Chang Lee, Ok-Jun Lee

**Affiliations:** 1 Department of Pathology, Chungbuk National University Hospital, Cheongju, Republic of Korea; 2 Department of Pathology, College of Medicine, Chungbuk National University, 1, Chungdae-Ro, Seowon-Gu, Cheongju, 28644 Republic of Korea; 3 Department of Pharmaceutical Engineering, Cheongju University, Cheongju, Republic of Korea; 4 College of Pharmacy, Wonkwang University, Iksan, Republic of Korea; 5 Department of Pharmacy, College of Pharmacy, Research Institute for Drug Development, Pusan National University, Busan, Republic of Korea; 6 College of Pharmacy, Chungbuk National University, Cheongju, Republic of Korea; 7 Department of Biochemistry, College of Medicine, Chungbuk National University, Cheongju, Republic of Korea; 8 Department of Internal Medicine, Chungbuk National University Hospital, Cheongju, Republic of Korea; 9 Department of Internal Medicine, College of Medicine, Chungbuk National University, Cheongju, Republic of Korea

**Keywords:** miR-29a, Carcinoembryonic antigen-related cell adhesion molecule 6 (CEACAM6), pH low insertion peptide (pHLIP), Lung adenocarcinoma, Tumor microenvironment

## Abstract

**Background:**

Non-coding microRNAs (miRNAs) play critical roles in tumor progression and hold great promise as therapeutic agents for multiple cancers. MicroRNA 29a (miR-29a) is a tumor suppressor miRNA that inhibits cancer cell growth and tumor progression in non-small cell lung cancer. Carcinoembryonic antigen-related cell adhesion molecule 6 (CEACAM6), which plays an important role in lung cancer progression, has been identified as a target of miR-29a. Here, we evaluated the therapeutic efficacy of a peptide vector capable of delivering miR-29a intracellularly using the acidic tumor microenvironment in a lung adenocarcinoma xenograft mouse model.

**Methods:**

A miRNA delivery vector was constructed by tethering the peptide nucleic acid form of miR-29a to a peptide with a low pH-induced transmembrane structure (pHLIP) to enable transport of the miRNAs across the plasma membrane. Tumor suppressive effects of pHLIP-miR29a on lung adenocarcinoma development in vivo were assessed using a BALB/c xenograft model injected with A549 cells.

**Results:**

Incubation of A549 cells with pHLIP-miR-29a at an acidic pH downregulated endogenous CEACAM6 expression and reduced cell viability. Intravenous injection of the mice with pHLIP-miR-29a inhibited tumor growth by up to 18.1%. Intraperitoneal injection of cisplatin reduced tumor volume by 29.9%. Combined pHLIP-miR-29a + cisplatin treatment had an additive effect, reducing tumor volume up to 39.7%.

**Conclusions:**

Delivery of miR-29a to lung adenocarcinoma cells using a pHLIP-mediated method has therapeutic potential as a unique cancer treatment approach.

**Supplementary Information:**

The online version contains supplementary material available at 10.1186/s12885-023-11352-w.

## Background

Lung cancer is the major cause of cancer-related death worldwide, with non-small cell lung cancer (NSCLC) accounting for approximately 80% of lung cancer cases [1]. Despite recent advances in treatment options, the prognosis of patients with NSCLC remains dismal [2]. Non-coding microRNAs (miRNAs) play key roles in the progression of lung and other cancers by regulating multiple biological processes, including tumorigenesis and cell growth and differentiation [3]. Members of the microRNA 29 (miR-29) family of miRNAs, comprising miR-29a, miR-29b, and miR-29c, act as tumor suppressors in several cancer types by regulating epigenetics, cell proliferation, apoptosis, and epithelial-mesenchymal transition [3, 4]. Overexpression of miR-29a limits cancer cell growth and tumor progression in subcutaneous models of NSCLC [5, 6].

We demonstrated previously that miR-29a acts as a tumor suppressor in NSCLC by targeting carcinoembryonic antigen-related cell adhesion molecule 6 (CEACAM6) [5]. Overexpression of CEACAM6 promotes cancer progression via its effects on cell proliferation, migration, and invasion, as well as tumor cell metastasis [7, 8]. CEACAM6 is overexpressed in nearly 70% of epithelial malignancies, including NSCLC and pancreatic, colon, breast, and gastric carcinomas [9–15]. In addition to *CEACAM6*, mir-29a also targets *LASP1* [6] and *CDC42* [16] and regulates proliferation, migration, and invasion of lung adenocarcinoma cells. Therefore, systemic delivery of miR-29a may be an effective strategy for treatment of lung cancer.

Overexpression of tumor suppressor miRNAs and inhibition of oncogenic miRNAs have shown therapeutic potential in model systems [17]. Moreover, the ability of small interfering RNAs (siRNAs) to silence oncogene expression via RNA interference (RNAi) suggests that they hold great promise as therapeutic agents for cancer [18]. Although the therapeutic potential of miRNA replacement therapy is substantial and many viral and non-viral vehicles have been designed [19]. Delivering miRNAs by viral vectors have proven unsuccessful as they elicit an immune response. Consequently, researchers have shifted their attention towards exploring various non-viral vectors [20]. Non-viral vectors are categorized into three groups: polymeric vectors, lipid-based carriers, and inorganic materials [21]. However, non-viral vectors exhibit low delivery efficiency and may cause toxicity [21–23]. A class of peptides known as pH low insertion peptides (pHLIPs) can be inserted into the cell membrane under acidic conditions through the formation of an inducible transmembrane α-helix, and can translocate membrane-impermeable molecules into cells [24, 25]. Because of the acidic tumor microenvironment, pHLIPs can target a variety of solid tumors and avoid systemic clearance by the liver [26]. Cheng et al*.* [27] demonstrated the therapeutic effect of miRNA silencing by tethering antisense oligomers against oncogenic miRNAs to a pHLIP for intracellular delivery. Specifically, Cheng et al*.* used peptide nucleic acids (PNAs) as antisense oligomer analogs. PNAs consist of nucleobases that are connected by intramolecular amide bonds. The backbone of PNAs is composed of peptide-like structure of repeated N-(2-aminoethyl)-glycine units. The lack of anionic phosphodiester groups increases the PNAs binding affinity for complementary nucleic acids. In addition, PNAs have high resistance to nucleases and proteases, and exhibit exceptional stability over a broad pH range [28].

Recently, we demonstrated the therapeutic effect of delivery of siCEACAM6 into cells in the acidic tumor microenvironment using a PNA form of CEACAM6-specific siRNA (siCEACAM6) as a peptide vector [29]. In the current study, we utilized the potent binding affinity of a PNA form of miR-29a to construct a pHLIP-fused tumor-targeting miRNA delivery vector and demonstrated the therapeutic effect of miR-29a delivery via the construct in a lung adenocarcinoma xenograft model.

## Methods

### Synthesis of PNA-pHLIP

PNA-pHLIP was generated according to the protocols described previously [29]. PNA versions of the miR-29a mimic (TAMRA–ooo-TAGCACCATCTGAAATCGGTTA-ooo-Cys) and a scrambled miRNA (scr; TAMRA–ooo-TCACAACCTCCTAGAAAGAGTAGA-ooo-Cys) were purchased from PANAGENE (Daejeon, South Korea). The PNA oligomer sequences including 11-amino-3,6,9-trioxaundecanoic acid (-ooo-) as a hydrophilic linker and cysteine were generated by solid-phase synthesis using an automatic synthesizer with Bts PNA monomers, which are proprietary PANAGENE building blocks for PNA oligomer synthesis. The oligomers were cleaved from the resin using an m-cresol:TFA (1:4) cocktail solution. TAMRA was exclusively conjugated to the PNA amino (N)-terminus. The PNA oligomers were purified by reversed-phase high performance liquid chromatography (RP-HPLC) and characterized by matrix-assisted laser desorption/ionization-time of flight mass spectrometry. The estimated melting temperatures of the miR-29a and scrambled miRNA PNA oligomers were 79°C and 75°C, respectively.

The pHLIP sequence AAEQNPIYWARYADWLFTTPLLLLDLALLVDADEGTCG was synthesized to generate the pHLIP-PNA constructs. The C-terminus of each PNA oligomer was conjugated to pHLIP through a disulfide bond. pHLIP and PNA (peptide:PNA, 1.5:1) were reacted overnight in the dark in a mixture of dimethylformamide (DMF)/Tris (pH 7.5). After conjugation, pHLIP-PNA was purified by RP-HPLC and characterized by matrix-assisted laser desorption/ionization-time of flight mass spectrometry. pHLIP-PNAs were quantified by UV/VIS spectrophotometry at 260 nm using the following extinction coefficients: 13,700 M^−1^ cm^−1^ (A), 6,600 M^−1^ cm^−1^ (C), 11,700 M^−1^ cm^−1^ (G), 8,800 M^−1^ cm^−1^ (T), 200 M^−1^ cm^−1^ (F, phenylalanine), and 32,300 M^−1^ cm^−1^ (TAMRA). After quantification, the pHLIP-PNAs were freeze-dried.

### Cell culture

The human lung adenocarcinoma cell line A549 and human colon adenocarcinoma cell line HT-29 were purchased from the American Type Culture Collection (Manassas, VA, USA). A549 and HT-29 cells were maintained in RPMI containing l-glutamine (2 mM), penicillin (100 U/ml), streptomycin (100 µg/ml), and 10% fetal bovine serum (FBS) and were cultured at 37°C in a 5% CO_2_ atmosphere. For all pH-controlled cell culture experiments, cells were incubated in complete culture medium with 10% FBS and buffered at pH 7.4 with 4-(2-hydroxyethyl)-1-piperazineethanesulfonic acid (HEPES) or pH 6.2 with 2-(N-morpholino)-ethanesulfonic acid (MES).

### Western blot analysis

Western blot analysis was conducted according to the protocols described previously [29]. Cells were incubated in culture medium supplemented with 10% FBS buffered at pH 7.4 with HEPES or pH 6.2 with MES and were treated with pHLIP-scr or pHLIP-miR-29a suspended in reaction buffer for 48 h. Proteins were extracted from the cells and separated by sodium dodecyl sulphate–polyacrylamide gel electrophoresis and transferred to polyvinylidene fluoride membranes (Thermo Fisher Scientific, Waltham, MA, USA). The membranes were blocked with 5% non-fat milk and then incubated with a mouse anti-CEACAM6 monoclonal antibody (9A6; Santa Cruz Biotechnology, Inc., Dallas, TX, USA), a rabbit anti-LASP1 monoclonal antibody (Abcam, Cambridge, MA, USA), a mouse anti-CDC42 monoclonal (B-8, Santa Cruz Biotechnology, Inc), or a mouse anti-GAPDH monoclonal antibody (6C5, Santa Cruz Biotechnology, Inc.), a mouse anti-β-actin monoclonal antibody (Sigma, St. Louis, MO, USA) as a loading control. The protein complexes were detected using enhanced chemiluminescence reagent (Thermo Fisher Scientific).

### Cell proliferation assay

The effects of pHLIP-miR-29a on the proliferation of A549 and HT-29 cells were measured using a Cell Proliferation Kit II (XTT) (Sigma-Aldrich, Inc., St. Louis, MO, USA). The cells (5 × 10^4^ /ml) were treated with pHLIP-miR-29a (0, 100, 250, or 500 nM) in 96-well plates for 48 h. Assays were performed in parallel using pHLIP-scr as a control. All treatments were performed at the indicated pH. After 48 h, the extent of cell growth was assessed using the Cell Proliferation Kit II (XTT). Briefly, XTT solution (50 μl) was added to each well and the cells were incubated for 2 h at 37 °C. Absorbance at 450 nm was then determined using a Lambda Bio-20 multiplate reader (Beckman Coulter, Brea, CA, USA). Cell proliferation was expressed as a percentage of that of control cells.

### In vivo tumor xenograft experiments

Animal experiments was conducted according to the protocols described previously [29]. All animal experiments were approved by the Institutional Animal Care and Use Committee of Korea Research Institute of Bioscience and Biotechnology. A549 cells (1.2 × 10^7^) were subcutaneously injected into the right flanks of 5-week-old male BALB/c athymic nude mice (*n* = 5 mice/group). Tumor growth (V) was determined by measuring the length (L), width (W), and height (H) with calipers and using the formula V = (L × W × H) × 0.5. Upon tumor formation (approximately 44.8 mm^3^), mice were intravenously injected with pHLIP-PNA constructs and/or intraperitoneally injected with cisplatin, as indicated. Mice were euthanized by carbon dioxide asphyxiation. Tumor volumes were measured every 2–3 days.

### Confocal microscopy

After euthanasia, tumors were harvested from mice, fixed in formalin, and paraffin embedded. The frozen tumor specimens were then cryosectioned into 2-μm sections. The sections were fixed with cold acetone, rinsed with phosphate-buffered saline (PBS), reacted with hydrogen peroxide to inactivate endogenous peroxidases, and then washed with PBS. The nuclei were stained using 4′,6-diamidino-2-phenylindole (DAPI). All washes to remove surface-bound pHLIP were performed using PBS at pH 7.4. Images were acquired using a LSM 710 META confocal microscope (Zeiss, Jena, Germany) and processed using Zen software (version 8.0; Zeiss).

### Histology and other techniques

Mice were euthanized and the tumors harvested, fixed in formalin, and paraffin embedded. The formalin-fixed, paraffin-embedded tumor specimens were sectioned (4 μm thickness) and then stained. Fully automated immunostaining was performed using a BenchMark XT autostainer (Ventana Medical Systems Inc., Tucson, AZ, USA). The following antibodies were used: monoclonal mouse anti-CC3 (1:100; BioCare Medical, Pacheco, CA, USA) and anti-Ki-67 (1:100; VP-K452; Vector Laboratories, Burlington, Canada). The number of positive cells per tumor area was quantified.

### Statistical analysis

Two-way analysis of variance was performed to compare the cell viability data from the three groups. For animal experiments, mouse and tumor weights for each group were compared using Student’s *t*-tests. Immunostaining data from each group were also compared using Student’s *t*-tests. Survival curves were estimated using the Kaplan–Meier method and compared using the log-rank test. *P* ≤ 0.05 was deemed statistically significant. All statistical tests were two-sided and performed using Prism 8 software (GraphPad, La Jolla, CA, USA).

## Results

### Generation and activity assessment of pHLIP-PNA miR-29a

To create the pHLIP-PNA miR-29a construct, the C-terminus of the PNA form of a miR-29a mimic was congugated to pHLIP. A control construct, pHLIP-scr, was also synthesized. A single isomer of 5-carboxytetramethylrhodamine (TAMRA) label was added to the PNA of the pHLIP-PNA oligomer. Verification of pHLIP-PNA oligomer production was conducted through RP-HPLC and mass spectrometry. (Supplementary Fig. 1).

**Fig. 1:**
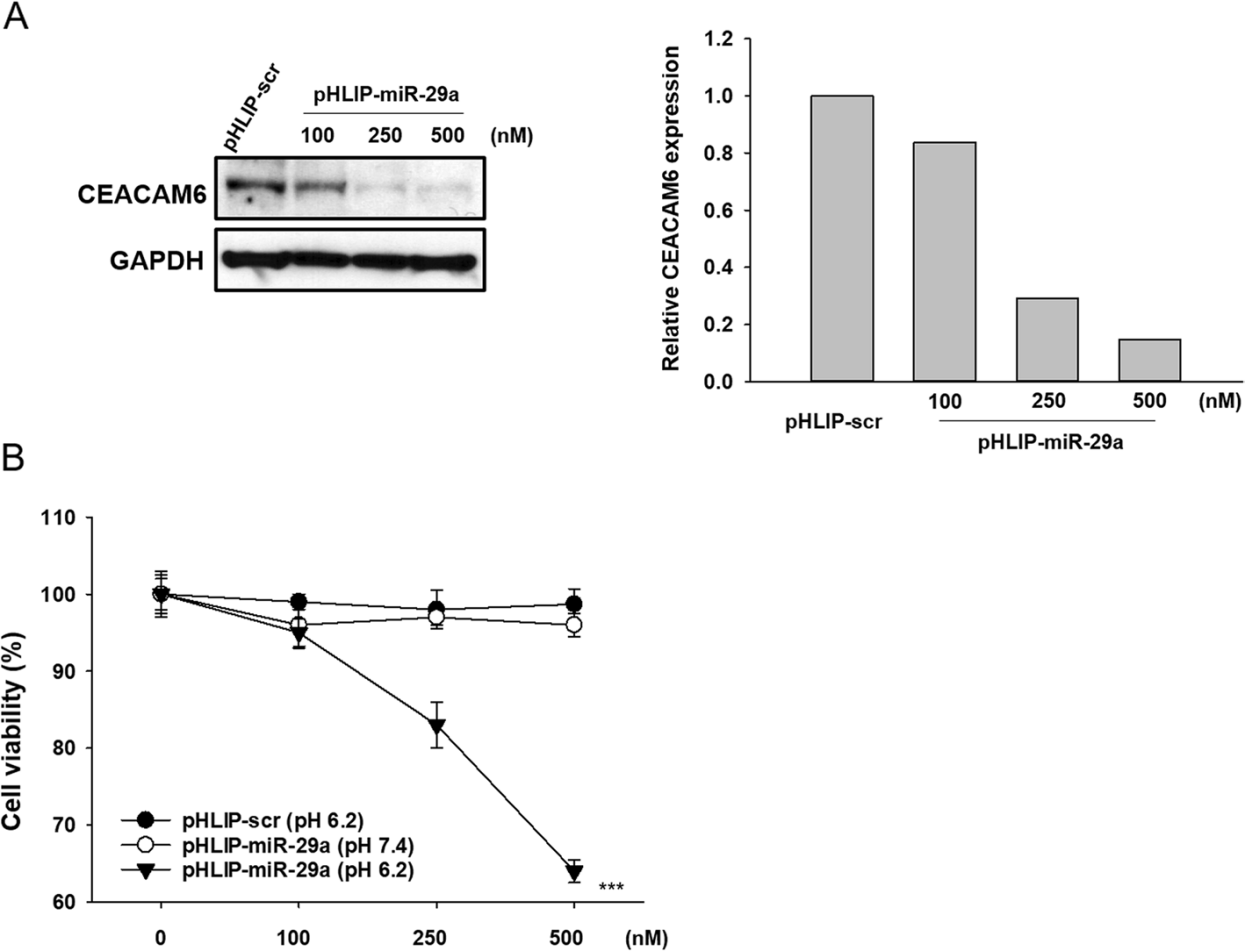
Activity of pHLIP-miR-29a targeting CEACAM6 in A549 cells. (**A**) Western blotting analysis of CEACAM6 protein levels in A549 cells incubated with pHLIP-miR-29a at pH 6.2. (**B**) Effects of pHLIP-miR-29a on the viability of A549 cells at neutral and acidic pH. Data are presented as the mean ± s.d. ***P < 0.001

To determine whether the pHLIP-PNA miR-29a conjugate could efficiently deliver miR-29a to cells and target CEACAM6, A549 lung adenocarcinoma cells were incubated for 48 h at pH 6.2 with 100, 250, or 500 nM pHLIP-miR-29a or pHLIP-scr as a control. Western blot analyses showed that pHLIP-miR-29a downregulated endogenous CEACAM6 expression at low pH in a dose-dependent manner, confirming the successful intracellular delivery of miR-29a and its inhibitory activity against CEACAM6 (Fig. 1a). In addition, pHLIP-mediated delivery of miR-29a reduced the viability of A549 cells in a dose-dependent manner at acidic pH, but not at neutral pH (Fig. 1b).

To evaluated the effect of pHLIP-miR-29a in other than lung cancer, we selected colorectal cancer, which is known for its typical expression of CEACAM6. In particular, we focused on the HT-29 cell line, which is known to express CEACAM6. Consistent with what was seen in A549 cells, pHLIP-miR-29a downregulated CEACAM6 expression in HT-29 cells (Supplementary Fig. 2a.) and reduced cell viability (Supplementary Fig. 2b) at low pH values in a dose-dependent manner. Taken together, these data indicate that pHLIP-miR-29a effectively translocated miR-29a to adenocarcinoma cells from various organs and inhibited protein expression of CEACAM6.

Furthermore, we investigated whether pHLIP-miR-29a inhibits other target proteins including LASP1 and CDC42 associated with lung adenocarcinoma cell proliferation. Consistent with the results obtained using CEACAM6, pHLIP-miR-29a downregulated LASP1 and CDC42 in A549 cells (Supplementary Fig. 3).

### Therapeutic efficacy of pHLIP-miR-29a in a lung cancer xenograft model

**Fig. 2:**
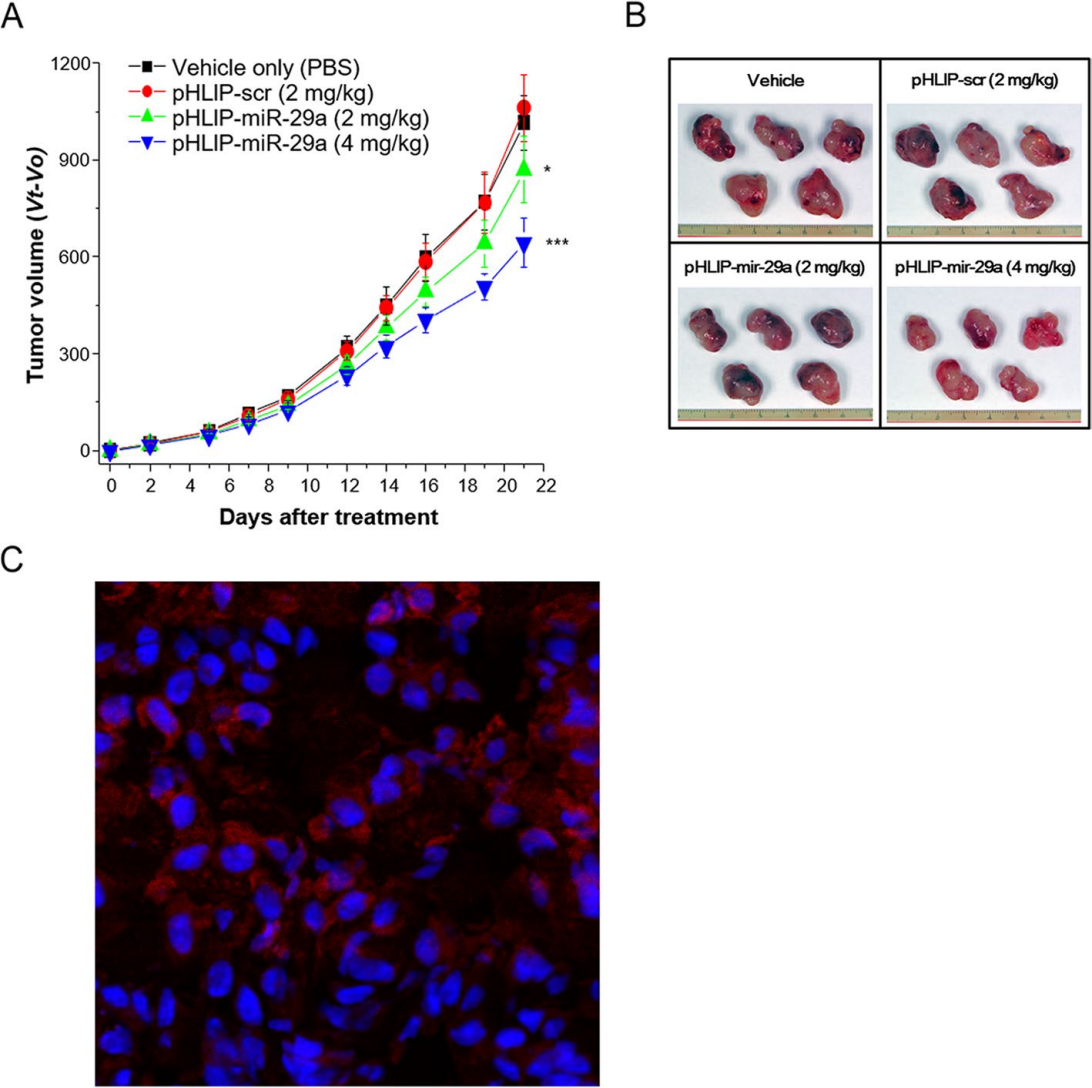
Administration of pHLIP-miR-29a delays lung tumor progression in an A549 xenograft mouse model. (**A**) Nude mice bearing A549 tumors were intravenously injected with pHLIP-miR-29a and tumor volumes were measured at the indicated days after treatment (*n* = 5 mice/group). Data are presented as the mean ± s.d. *P < 0.05; ***P < 0.001. (**B**) Representative images of tumors collected from nude mice 3 weeks after injection of pHLIP-scr or pHLIP-miR-29a. (**C**) Representative confocal image of A549 cells incubated with labelled pHLIP-miR-29a. Red, PNA-TAMRA; blue, nucleus

Next, we examined the effects of pHLIP-miR-29a delivery on tumor development in vivo in a lung adenocarcinoma xenograft model. For tumor induction, BALB/c nude mice were subcutaneously injected with A549 cells. Two weeks after injection of cancer cell suspension, the mice were randomized into three groups and tail-vein injected with pHLIP-miR-29a, PBS as a negative control, or pHLIP-scr as a vector control. To determine the optimal dose for injection, two groups of mice (*n* = 5 mice/group) were injected twice a week with different doses of pHLIP-miR-29a (2 and 4 mg/kg) for 3 weeks. The mice were sacrificed after 3 weeks of treatment. Tumor volumes of mice treated with pHLIP-miR-29a were significantly smaller than those of control mice treated with pHLIP-scr (Fig. 2a and 2b). Specifically, at 2 mg/kg the tumors of the pHLIP-miR-29a-treated mice were 17.6% smaller than those of mice treated with 2 mg/kg pHLIP-scr (*P* = 0.03). At 4 mg/kg, pHLIP-miR-29a treatment reduced tumor size by 40.9% compared with treatment with pHLIP-scr (*P* < 0.001). The ability of pHLIP-miR-29a to deliver miR-29a to lung adenocarcinomas in vivo was assessed by confocal imaging of the isolated tumor tissues. Strong fluorescent TAMRA signals were observed on the surfaces of tumor cells from the pHLIP-miR-29a-treated mice, indicating efficient in vivo intracellular delivery of miR-29a (Fig. 2c). Mice treated with pHLIP-miR-29a exhibited no clinical signs of distress, body weight changes (Supplementary Fig. 4a), or damage to organs, including the kidney, liver, and heart (Supplementary Fig. 4b).

**Fig. 3:**
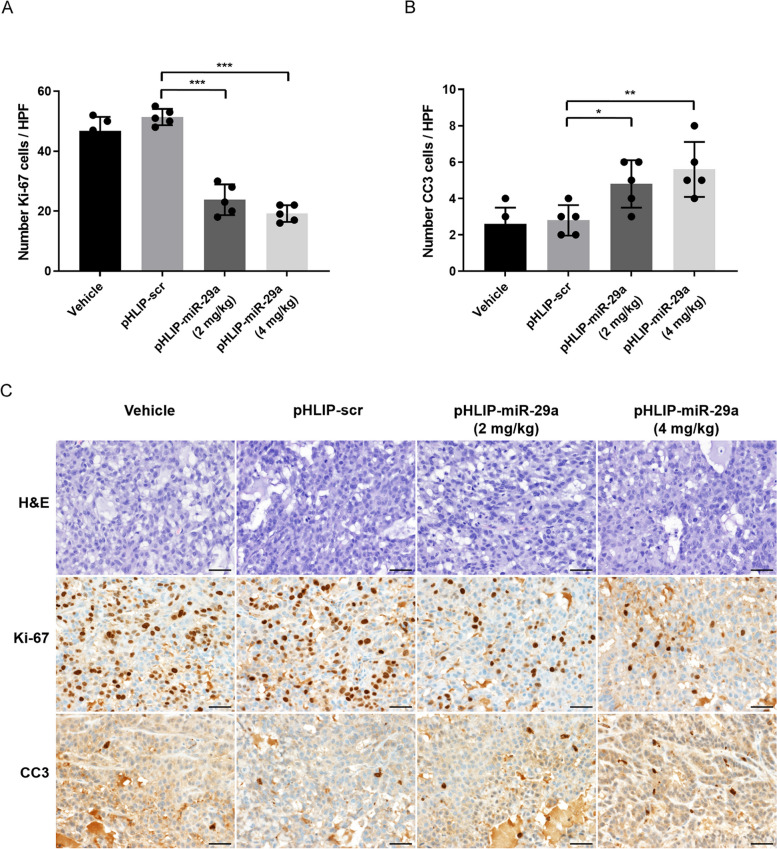
Effects of pHLIP-miR-29a on tumor cell proliferation and apoptosis in a lung cancer xenograft mouse model. (A, B) Quantification of dividing cells labelled by Ki-67 staining (**A**) and apoptotic cells labelled by CC3 staining (**B**) in tumors from mice injected with pHLIP-miR-29a or pHLIP-scr (*n* = 5 tumors/group). Data are presented as the mean ± s.d. *P < 0.05; **P < 0.01; ***P < 0.001. (**C**) Representative histological and immunohistochemical analyses of lung tumor sections from mice treated with vehicle, pHLIP-scr, or pHLIP-miR-29a (2 mg/kg or 4 mg/kg). Original magnification × 400; scale bar = 50 µm

To investigate the mechanisms underlying tumor regression in the pHLIP-miR-29a-treated mice, tumor tissues were harvested and stained for markers of proliferation and apoptosis. Tumors from pHLIP-miR-29a-treated mice had significantly fewer Ki-67-positive cells than those from pHLIP-scr-treated mice (*P* < 0.001) As shown in Fig. 3A and 3C, the prevalence of Ki-67-positive cells was 51.4% in the pHLIP-scr-treated mice and 23.8% in the mice treated with 2 mg/kg pHLIP-miR-29a. In addition, the percentage of cells positive for cleaved caspase 3 (CC3), a marker of apoptosis, was significantly higher in the pHLIP-miR-29a-treated group than that in the pHLIP-scr-treated group (*P* = 0.02; (Fig. 3b and 3c). Overall, these results suggest that delivery of miR-29a suppressed tumor growth in the lung cancer xenograft mouse model by inhibiting tumor cell proliferation and promoting apoptosis.

### Therapeutic responses to concurrent treatment with pHLIP-miR-29a and cisplatin

**Fig. 4:**
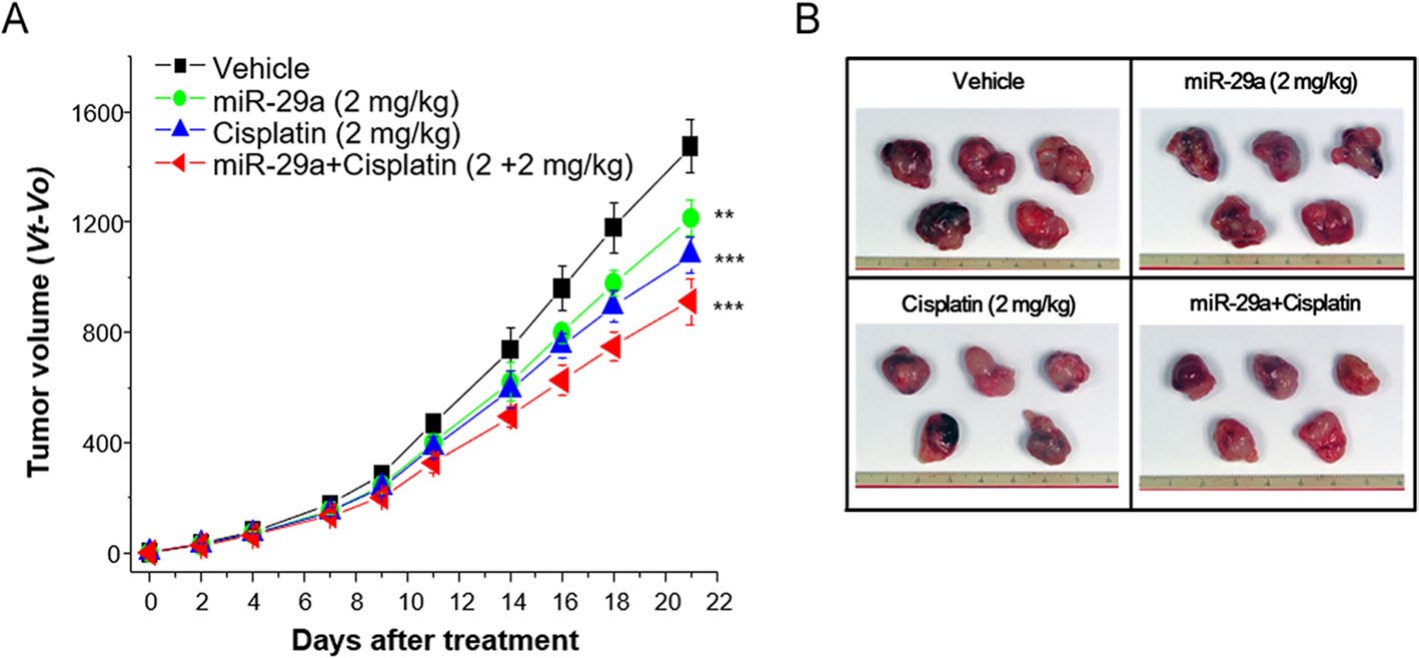
Therapeutic responses in a lung cancer xenograft mouse model after concurrent treatment with miR-29a and cisplatin. (**A**) Nude mice bearing A549 tumors were intravenously injected with pHLIP-miR-29a and/or cisplatin and tumor volumes were measured at the indicated days after treatment (*n* = 5 mice/group). Data are presented as the mean ± s.d. **P < 0.01, ***P < 0.001. (**B**) Representative images of tumors collected from nude mice 3 weeks after injection of pHLIP-miR-29a and/or cisplatin

As cisplatin is the standard of lung cancer treatment, we compared the anti-tumor effects of cisplatin and pHLIP-miR-29a individually and in combination to assess the therapeutic effects of concurrent chemotherapy and miR-29a treatment. After tumor initiation with A549 cells, mice were randomly assigned to the following treatment groups (*n* = 5 mice/group): (i) vehicle, (ii) pHLIP-miR-29a, (iii) cisplatin, and (iv) pHLIP-miR-29a + cisplatin. Based on the in vivo results, a dose of 2 mg/kg pHLIP-miR-29a was selected for the combination treatment. Two weeks after cell line grafting, pHLIP-miR-29a was tail-vein injected into the mice and a dose of 2 mg/kg of cisplatin was intraperitoneally injected twice per week for 3 weeks. As shown in Fig. 4, the mean tumor volume of mice in the pHLIP-miR-29a treatment group was 18.1% smaller than that in the vehicle-treated group after 3 weeks of systemic therapy (*P* = 0.004). Cisplatin treatment reduced the tumor volume by 29.9% (*P* < 0.001), whereas pHLIP-miR-29a + cisplatin treatment reduced the tumor volume by 39.7% (*P* < 0.001). These results indicate that systemically delivered miR-29a and cytotoxic chemotherapy had additive anti-tumor effects in vivo. None of the mice in the therapeutic experiments experienced overt body weight loss (Supplementary Fig. 5a) or organ damage (Supplementary Fig. 5b) throughout the treatment period.

**Fig. 5:**
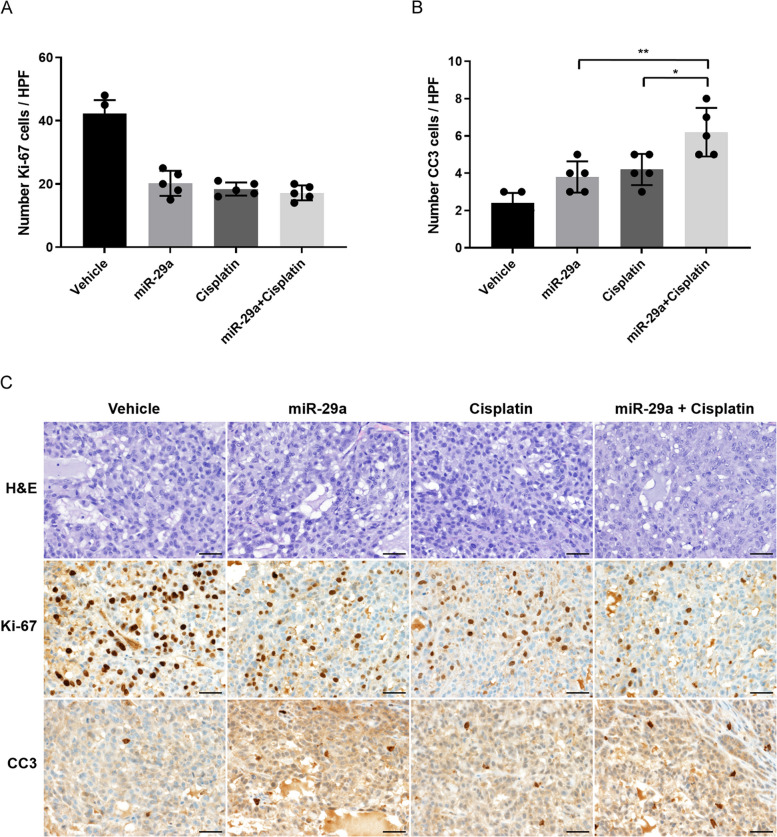
Combined effects of pHLIP-miR-29a and cisplatin on tumor cell proliferation and apoptosis in a lung cancer xenograft mouse model. (A, B) Quantification of dividing cells labelled by Ki-67 staining (**A**) and apoptotic cells labelled by CC3 staining (**B**) in tumors from injected with pHLIP-miR-29a and/or cisplatin (*n* = 5 tumors/group). Data are presented as the mean ± s.d. *P < 0.05; **P < 0.01. (**C**) Representative histological and immunohistochemical analyses of lung tumor sections from mice treated with vehicle, pHLIP-miR-29a, cisplatin, or pHLIP-miR-29a + cisplatin. Original magnification × 400; scale bar = 50 µm

To assess further the biologic effects of combined systemic miR-29a and cisplatin treatments, tumor samples were stained for markers of proliferation and apoptosis. As shown in Fig. 5A and 5C, cell proliferation in tumors from mice treated with pHLIP-miR-29a + cisplatin (17.2%) did not differ significantly from that in tumors from mice treated with pHLIP-miR-29a alone (20.2%; *P* = 0.185) or cisplatin alone (18.4%; *P* = 0.421). However, tumors from mice treated with pHLIP-miR-29a + cisplatin showed significantly higher numbers of CC3-positive cells than those from mice treated with pHLIP-miR-29a alone (*P* = 0.009) or cisplatin alone (*P* = 0.02), as shown in Fig. 5B and 5C. These results indicate that combination treatment with pHLIP-miR-29a and cisplatin had an additive therapeutic effect based on tumor volume measurements and immunohistochemical staining of apoptosis markers.

## Discussion

We found that systemic administration of a pHLIP-miR-29a conjugate resulted in successful delivery of tumor suppressive miR-29a to lung adenocarcinoma cells in mice by targeting the acidic tumor microenvironment. At acidic pH, pHLIP forms an α-helix that inserts into the lipid bilayer, making it an attractive targeting moiety for selective labelling and tracing of acidic tissues in vivo [30]. As acidosis is a general property of the tumor microenvironment [26], pHLIP localizes to tumors. In the current study, we tethered a PNA form of miR-29a to the C-terminus of pHLIP, thereby ensuring its delivery across the plasma membrane. The PNA and peptide were linked by a disulfide bond that was reduced in the cytosol, resulting in intracellular delivery of the free PNA.

The pHLIP-mediated delivery of the miR-29a mimic inhibited CEACAM6 protein expression in A549 lung adenocarcinoma cells and reduced cell proliferation. The expression of LASP1 and CDC42 proteins were also downregulated by pHLIP-miR-29a. In vivo delivery of pHLIP-miR-29a inhibited the growth of lung adenocarcinoma tumors. These findings were consistent with the results of our previous study in which injection of mice with A549 cells expressing miR-29a inhibited the tumor growth in a lung adenocarcinoma mouse model [5].

MiR-29a appears to exert its tumor suppressor effects by affecting the regulation of multiple biological processes [5, 6, 16, 31, 32]. Regarding epigenetic modulation, members of the miR-29 family are reported to repress the activities of DNA methyltransferases 3A and 3B, as well as DNA demethylases TET1 and TDG, which have opposing functions in the control of DNA methylation [32]. Accordingly, miR-29 may suppress tumorigenesis by protecting against changes in the existing DNA methylation status and by acting as a stabilizer of DNA methylation. Furthermore, Liu et al*.* [31] showed that overexpression of miR-29a inhibits the proliferation of lung cancer cells and increases their chemosensitivity to cisplatin by targeting NRAS. MiR-29a also plays a significant role in promoting apoptosis via several effectors including MCL-1 [33], KDM5B [34], QKI-6 [35], MMP2 [36], and TNFR1 [37] in various cancers. In our current study, treatment with miR-29a increased expression of the apoptosis marker CC3, suggesting miR-29a may also promote apoptosis of tumor cells.

In the mouse lung adenocarcinoma xenograft model, the anti-tumor effects of the combination of miR-29a and the chemotherapeutic drug cisplatin were superior to those of either treatment alone. Previous studies have found that reduced levels of miR-29 lead to increased resistance of ovarian cancer cells to cisplatin [38] and miR-29a sensitizes lung cancer cells to cisplatin [31]. The results presented in the current study suggest that miR-29a delivery may improve the efficacy of cisplatin treatment by inhibiting CEACAM6-mediated chemoresistance pathways. Overexpression of CEACAM6 increases gemcitabine chemoresistance in pancreatic adenocarcinoma cells by modulating Akt activity in a c-Src-dependent manner and inhibition of CEACAM6 restores the paclitaxel sensitivity of lung adenocarcinomas [39, 40]. Therefore, RNA therapy could be combined with chemotherapy to improve the efficacy of existing cancer treatments by modulating the expression of genes involved in chemoresistance pathways.

None of the mice in our experiments exhibited any overt signs of toxicity. The tumor-targeting property of pHLIP ensured the miR-29a delivery was limited to tumor tissues; however, additional studies are required to delineate the impact of miR-29a delivery on normal tissues and the tumor microenvironment.

RNA-based therapies hold promise to expand the range of druggable targets from proteins to RNAs and the genome. Lipid nanoparticle-formulated siRNA delivery is safe and well-tolerated in first-in-human clinical trials and in some metastatic cancer cases elicited complete responses [41]. In addition, intravenously administered antisense oligonucleotides targeting *KRAS* mRNA have been tested in phase I clinical trials for treatment of NSCLC and other advanced solid tumors [18]. Development of vehicles capable of efficient intracellular delivery is required to enable the extension of small RNA therapies to other major cancer types.

## Conclusions

In summary, the present study demonstrated the efficacy of small RNA-based therapy in a lung adenocarcinoma xenograft model. A pHLIP vector was used to improve tumor-targeting and mediate the successful delivery of a tumor suppressor miRNA. The effective delivery of therapeutic miRNAs to lung cancer cells using the pHLIP peptide increases the prospect of using small RNAs for cancer therapy.

### Supplementary Information

**Additional file 1.** **Supplementary Figure 1.** RP-HPLC elution profiles of a PNA (siCEACAM6) and pHLIP reaction mixture.

**Additional file 2.** **Supplementary Figure 2.** Activity of pHLIP-miR-29a targeting CEACAM6 in HT-29 colon adenocarcinoma cells. (A) Western blotting analysis of CEACAM6 protein levels in HT-29 cells incubated with the indicated concentration of pHLIP-miR-29a at pH 6.2. (B) Effects of pHLIP-miR-29a on the viability of HT-29 cells at neutral and acidic pH. Data are presented as the mean ± s.d. ***P < 0.001.

**Additioanal file 3.** **Supplementary Figure 3.** Activity of pHLIP-miR-29a targeting LASP1 and CDC42 in A549 cells. Western blotting analysis of LASP1 and CDC42 protein levels in A549 cells incubated with pHLIP-miR-29a at pH 6.2.

**Additional file 4.** **Supplementary Figure 4.** Toxicity assessment of intravenously administered pHLIP-miR-29a. (A) Changes in the body weights of experimental mice during the treatment period (*n* = 5 mice/group). Data are presented as the mean ± s.d. (B) Representative histological analyses of kidney, liver, and heart specimens collected from mice treated with pHLIP-miR-29a or pHLIP-scr. All sections reveal an absence of microscopic changes associated with toxicity in pHLIP-miR-29a-treated mice. Original magnification ×400; scale bar = 50 µm.

**Additional file 5.** **Supplementary Figure 5.** Toxicity assessment of combination treatment with pHLIP-miR-29a and cisplatin. (A) Changes in the body weights of experimental mice during the treatment period (*n* = 5 mice/group). Data are presented as the mean ± s.d. (B) Representative histological analysis of kidney, liver, and heart specimens collected from mice treated with pHLIP-miR-29a or pHLIP-miR-29a+cisplatin. All sections revealed an absence of microscopic changes associated with toxicity in pHLIP-miR-29a-treated and pHLIP-miR-29a+cisplatin-treated animals. Original magnification ×400; scale bar = 50 µm.

**Additional file 6.** **Supplementary Table 1.** The tumor volume of each animal in Figure 2A.

**Additional file 7.** **Supplementary Table 2.** The tumor volume of each animal in Figure 4A.

**Additional file 8.** Raw images.

## Data Availability

The data supporting the conclusions of this article are included in the article.

## Abbreviations

NSCLC: Non-small cell lung cancer
miRNAs: MicroRNAs
miR-29: MicroRNA 29
CEACAM6: Carcinoembryonic antigen-related cell adhesion molecule 6
siRNAs: Small interfering RNAs
RNAi: RNA interference
pHLIPs: PH low insertion peptides
PNAs: Peptide nucleic acids
siCEACAM6: CEACAM6-specific siRNA
RP-HPLC: Reversed-phase high performance liquid chromatography
CC3: Cleaved caspase 3
