# Supplementary figures and images for: MicroRNA 29a therapy for CEACAM6-expressing lung adenocarcinoma
Source: BMC Cancer. 2023 Sep 8;23:843. doi: 10.1186/s12885-023-11352-w (PMC10492333; doi:10.1186/s12885-023-11352-w)

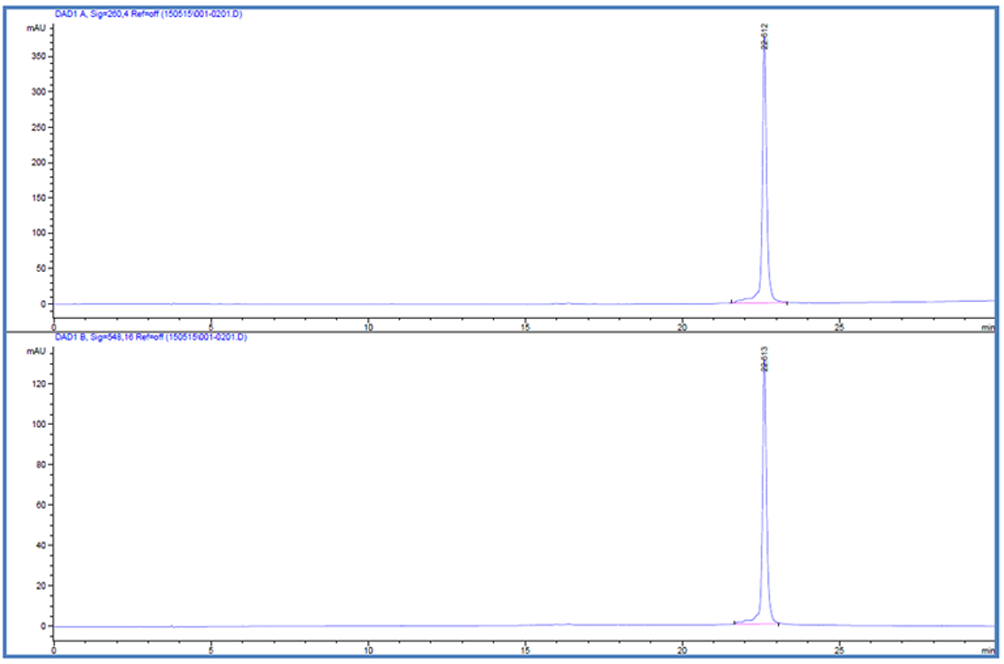

Supplement: Supplementary file 1 — Additional file 1. Supplementary Figure 1. RP-HPLC elution profiles of a PNA (siCEACAM6) and pHLIP reaction mixture. [file 12885_2023_11352_MOESM1_ESM.tif]

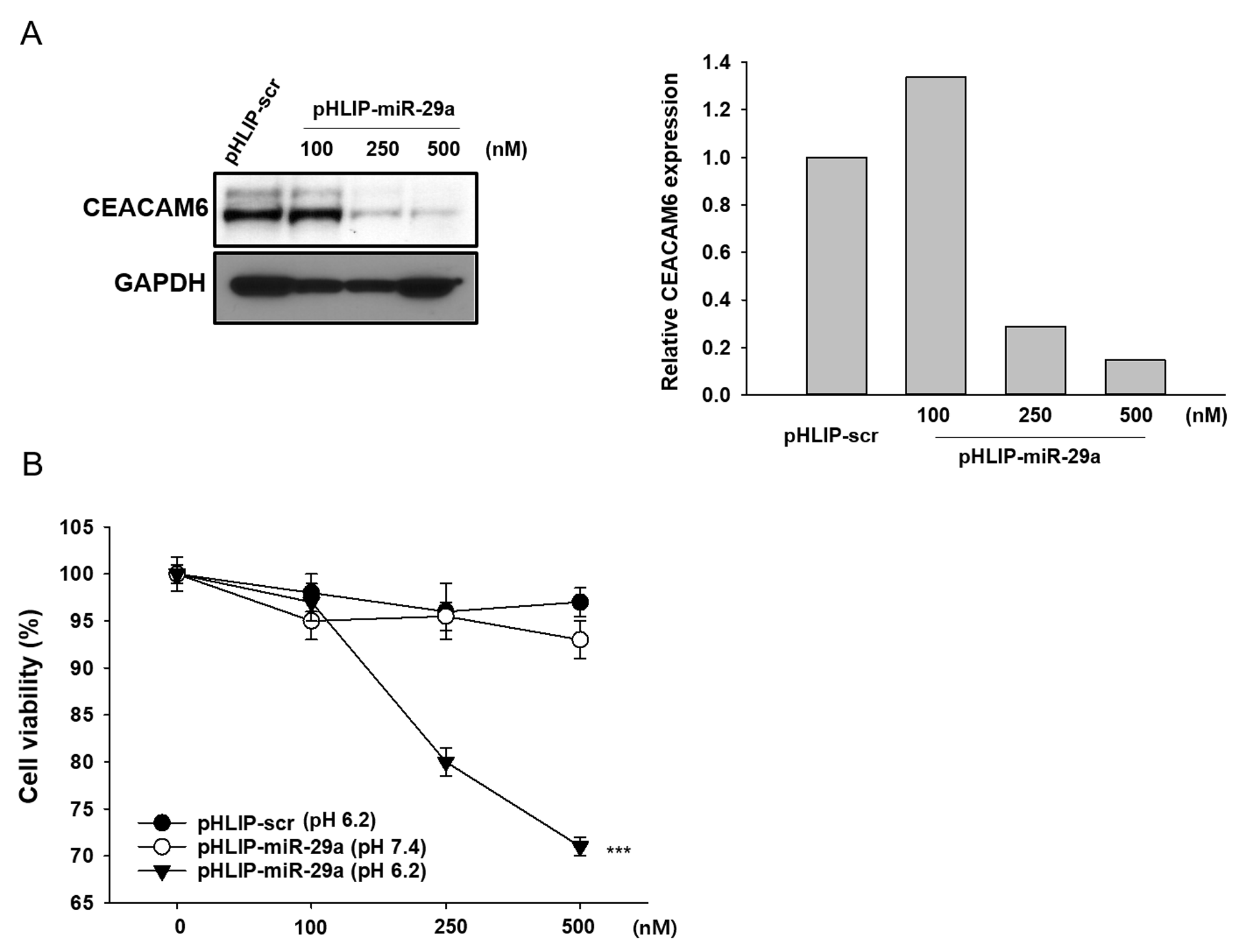

Supplement: Supplementary file 2 — Additional file 2. Supplementary Figure 2. Activity of pHLIP-miR-29a targeting CEACAM6 in HT-29 colon adenocarcinoma cells. (A) Western blotting analysis of CEACAM6 protein levels in HT-29 cells incubated with the indicated concentration of pHLIP-miR-29a at pH 6.2. (B) Effects of pHLIP-miR-29a on the viability of HT-29 cells at neutral and acidic pH. Data are presented as the mean ± s.d. ***P < 0.001. [file 12885_2023_11352_MOESM2_ESM.tif]

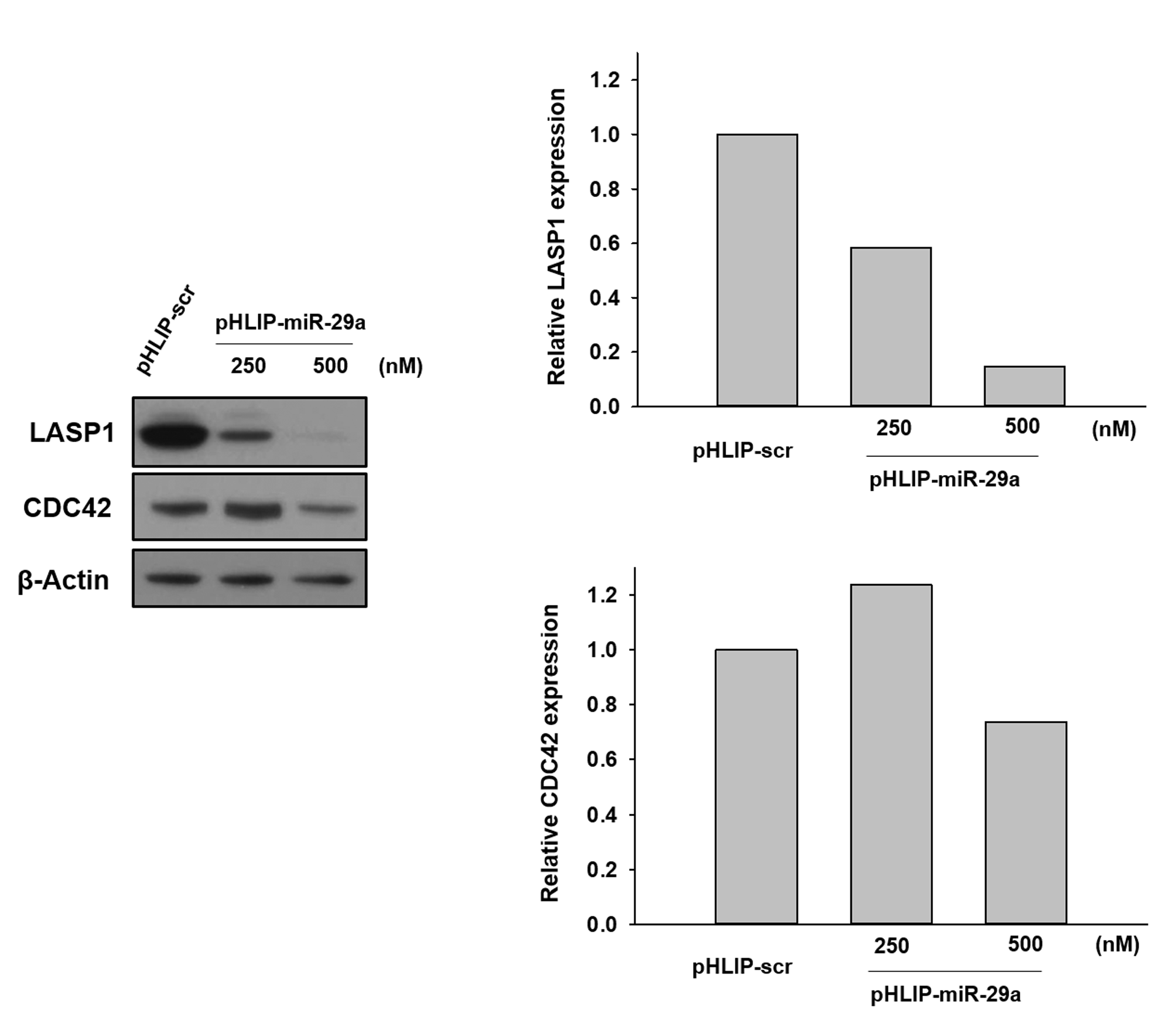

Supplement: Supplementary file 3 — Additioanal file 3. Supplementary Figure 3. Activity of pHLIP-miR-29a targeting LASP1 and CDC42 in A549 cells. Western blotting analysis of LASP1 and CDC42 protein levels in A549 cells incubated with pHLIP-miR-29a at pH 6.2. [file 12885_2023_11352_MOESM3_ESM.tif]

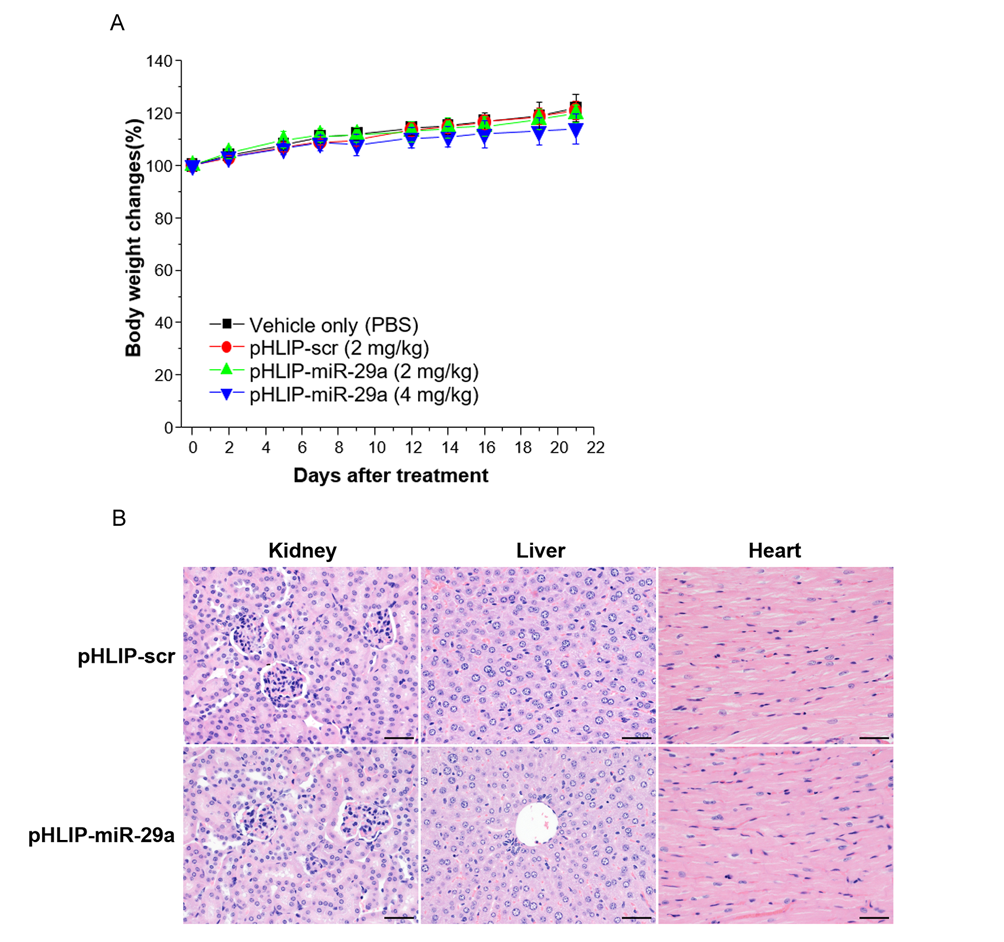

Supplement: Supplementary file 4 — Additional file 4. Supplementary Figure 4. Toxicity assessment of intravenously administered pHLIP-miR-29a. (A) Changes in the body weights of experimental mice during the treatment period (n = 5 mice/group). Data are presented as the mean ± s.d. (B) Representative histological analyses of kidney, liver, and heart specimens collected from mice treated with pHLIP-miR-29a or pHLIP-scr. All sections reveal an absence of microscopic changes associated with toxicity in pHLIP-miR-29a-treated mice. Original magnification ×400; scale bar = 50 µm. [file 12885_2023_11352_MOESM4_ESM.tif]

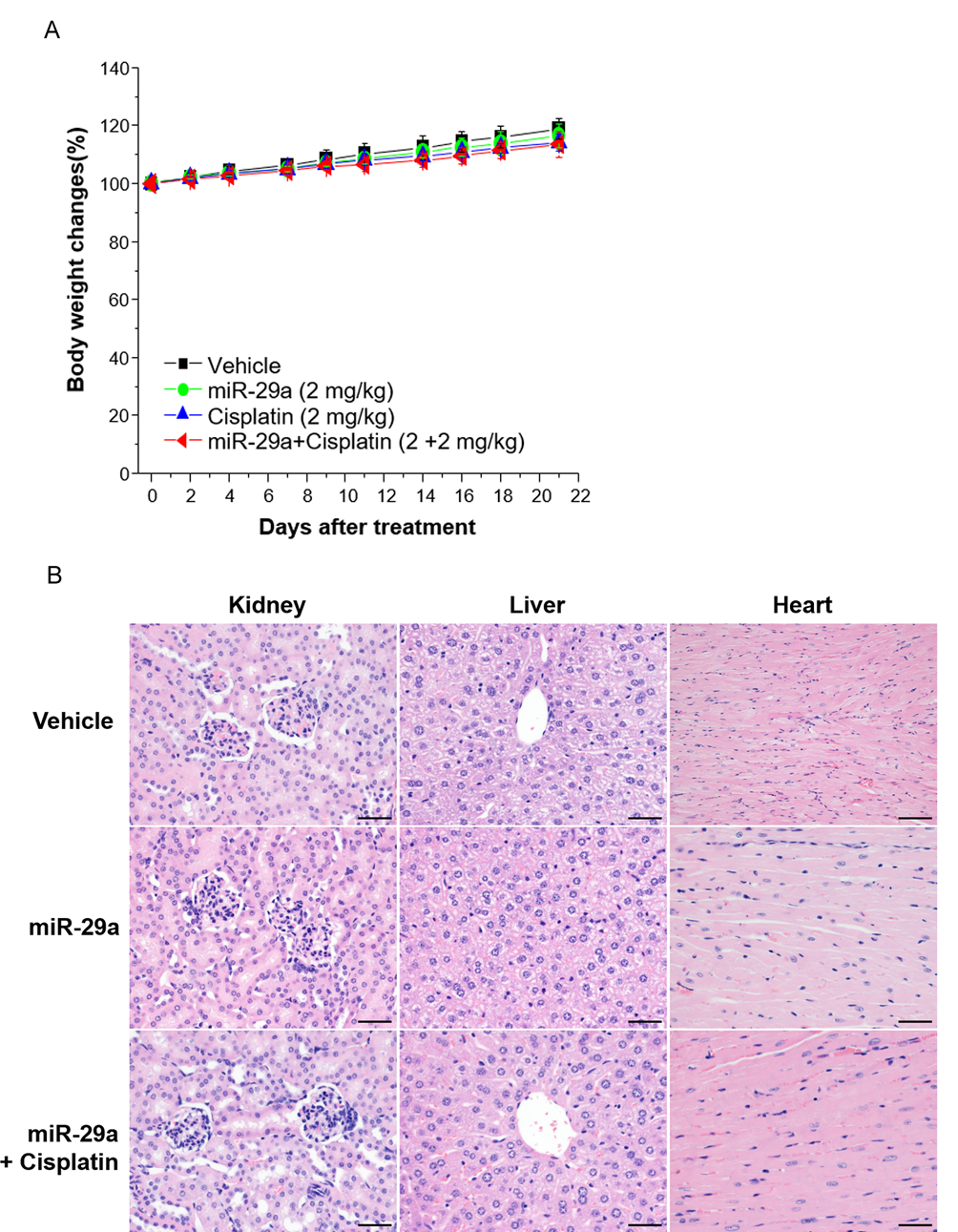

Supplement: Supplementary file 5 — Additional file 5. Supplementary Figure 5. Toxicity assessment of combination treatment with pHLIP-miR-29a and cisplatin. (A) Changes in the body weights of experimental mice during the treatment period (n = 5 mice/group). Data are presented as the mean ± s.d. (B) Representative histological analysis of kidney, liver, and heart specimens collected from mice treated with pHLIP-miR-29a or pHLIP-miR-29a+cisplatin. All sections revealed an absence of microscopic changes associated with toxicity in pHLIP-miR-29a-treated and pHLIP-miR-29a+cisplatin-treated animals. Original magnification ×400; scale bar = 50 µm. [file 12885_2023_11352_MOESM5_ESM.tif]
