# Supplementary material for: MicroRNA 29a therapy for CEACAM6-expressing lung adenocarcinoma
Source: BMC Cancer. 2023 Sep 8;23:843. doi: 10.1186/s12885-023-11352-w (PMC10492333; doi:10.1186/s12885-023-11352-w)
Supplement: Supplementary file 6 — Additional file 6. Supplementary Table 1. The tumor volume of each animal in Figure 2A. [file 12885_2023_11352_MOESM6_ESM.docx]

Supplementary Table 1. The tumor volume of each animal in Figure 2A

| Group | Doses  (mg/kg) | Days | Animal number | | | | | Mean | Standard deviation | P-value  (t-TEST) |
| --- | --- | --- | --- | --- | --- | --- | --- | --- | --- | --- |
|  |  |  | 1 | 2 | 3 | 4 | 5 |  |  |  |
| Vehicle only  (PBS, pH7.4) | 0 | 0805 Day 0  Day 0 – Day 0 | 41.1  0.0 | 42.4  0.0 | 42.4  0.0 | 43.6  0.0 | 44.0  0.0 | 42.7  0.0 | 1.1  0.0 |  |
|  |  | 0807 Day 2  Day 2 – Day 0 | 60.7  19.6 | 62.4  20.0 | 65.7  23.3 | 64.8  21.2 | 67.6  23.6 | 64.2  21.5 | 2.7  1.8 |  |
|  |  | 0810 Day 5  Day 5 – Day 0 | 94.4  53.3 | 100.3  57.9 | 107.6  65.2 | 101.6  58.0 | 111.1  67.1 | 103.0  60.3 | 6.5  5.7 |  |
|  |  | 0812 Day 7  Day 7 – Day 0 | 146.6  105.5 | 148.1  105.7 | 150.7  108.3 | 162.4  118.8 | 170.6  126.6 | 155.7  113.0 | 10.4  9.4 |  |
|  |  | 0814 Day 9  Day 9 – Day 0 | 190.0  148.9 | 209.0  166.6 | 206.7  164.3 | 218.4  174.8 | 226.2  182.2 | 210.1  167.4 | 13.6  12.5 |  |
|  |  | 0817 Day 12  Day 12 – Day 0 | 299.5  258.4 | 370.9  328.5 | 352.8  310.4 | 381.6  338.0 | 398.8  354.8 | 360.7  318.0 | 38.1  37.0 |  |
|  |  | 0819 Day 14  Day 14 – Day 0 | 400.9  359.8 | 520.4  478.0 | 462.1  419.7 | 517.5  473.9 | 550.7  506.7 | 490.3  447.6 | 59.3  58.3 |  |
|  |  | 0821 Day 16  Day 16 – Day 0 | 534.3  493.2 | 674.5  632.1 | 594.6  552.2 | 689.4  645.8 | 704.0  660.0 | 639.4  596.7 | 72.4  71.4 |  |
|  |  | 0824 Day 19  Day 19 – Day 0 | 745.9  704.8 | 749.8  707.4 | 763.8  721.4 | 860.4  816.8 | 943.6  899.6 | 812.7  770.0 | 86.9  85.9 |  |
|  |  | 0826 Day 21  Day 21 – Day 0 | 1059.0  1017.9 | 1005.9  963.5 | 958.0  915.6 | 1081.3  1037.7 | 1186.4  1142.4 | 1058.1  1015.4 | 86.2  85.6 |  |

| Group | Doses  (mg/kg) | Days | Animal number | | | | | Mean | Standard deviation | P-value  (t-TEST) |
| --- | --- | --- | --- | --- | --- | --- | --- | --- | --- | --- |
|  |  |  | 1 | 2 | 3 | 4 | 5 |  |  |  |
| pHLlP-scr | 2 | 0805 Day 0  Day 0 – Day 0 | 40.5  0.0 | 41.7  0.0 | 42.5  0.0 | 43.6  0.0 | 44.8  0.0 | 42.6  0.0 | 1.7  0.0 |  |
|  |  | 0807 Day 2  Day 2 – Day 0 | 60.9  20.4 | 63.7  22.0 | 61.1  18.6 | 64.8  21.2 | 65.1  20.3 | 63.1  20.5 | 2.0  1.3 |  |
|  |  | 0810 Day 5  Day 5 – Day 0 | 88.1  47.6 | 106.4  64.7 | 96.8  54.3 | 101.3  57.7 | 107.0  62.2 | 99.9  57.3 | 7.8  6.7 |  |
|  |  | 0812 Day 7  Day 7 – Day 0 | 143.3  102.8 | 143.5  101.8 | 142.1  99.6 | 141.8  98.2 | 165.2  120.4 | 147.2  104.6 | 10.1  9.0 |  |
|  |  | 0814 Day 9  Day 9 – Day 0 | 206.2  165.7 | 197.0  155.3 | 186.3  143.8 | 200.3  156.7 | 222.0  177.2 | 202.4  159.7 | 13.1  12.5 |  |
|  |  | 0817 Day 12  Day 12 – Day 0 | 383.5  343.0 | 361.6  319.9 | 306.0  263.5 | 348.5  304.9 | 350.0  305.2 | 349.9  307.3 | 28.3  29.0 |  |
|  |  | 0819 Day 14  Day 14 – Day 0 | 533.4  492.9 | 503.9  462.2 | 457.7  415.2 | 488.1  444.5 | 436.5  391.7 | 483.9  441.3 | 38.1  39.5 |  |
|  |  | 0821 Day 16  Day 16 – Day 0 | 664.8  624.3 | 626.6  584.9 | 560.0  517.5 | 699.3  655.7 | 586.3  541.5 | 627.4  584.8 | 56.6  57.0 |  |
|  |  | 0824 Day 19  Day 19 – Day 0 | 874.2  833.7 | 844.9  803.2 | 714.2  671.7 | 909.3  865.7 | 707.1  662.3 | 809.9  767.3 | 93.5  94.3 |  |
|  |  | 0826 Day 21  Day 21 – Day 0 | 1217.7  1177.2 | 1122.7  1081.0 | 967.0  924.5 | 1177.2  1133.6 | 1030.9  986.1 | 1103.1  1060.5 | 103.4  104.2 |  |

| Group | Doses  (mg/kg) | Days | Animal number | | | | | Mean | Standard deviation | P-value  (t-TEST) |
| --- | --- | --- | --- | --- | --- | --- | --- | --- | --- | --- |
|  |  |  | 1 | 2 | 3 | 4 | 5 |  |  |  |
| pHLlP-miR-29a | 2 | 0805 Day 0  Day 0 – Day 0 | 40.0  0.0 | 41.6  0.0 | 43.0  0.0 | 43.4  0.0 | 45.5  0.0 | 42.7  0.0 | 2.1  0.0 |  |
|  |  | 0807 Day 2  Day 2 – Day 0 | 61.1  21.1 | 59.9  18.3 | 60.6  17.6 | 62.8  19.4 | 63.8  18.3 | 61.6  18.9 | 1.6  1.4 | 0.09814 |
|  |  | 0810 Day 5  Day 5 – Day 0 | 91.4  51.4 | 93.5  51.9 | 93.5  50.5 | 97.9  54.5 | 95.9  50.4 | 94.4  51.7 | 2.5  1.7 | 0.11136 |
|  |  | 0812 Day 7  Day 7 – Day 0 | 125.0  85.0 | 127.2  85.6 | 132.4  89.4 | 137.3  93.9 | 150.7  105.2 | 134.5  91.8 | 10.2  8.3 | 0.04862 |
|  |  | 0814 Day 9  Day 9 – Day 0 | 160.7  120.7 | 177.8  136.2 | 178.1  135.1 | 192.2  148.8 | 202.2  156.7 | 182.2  139.5 | 15.8  13.8 | 0.04131 |
|  |  | 0817 Day 12  Day 12 – Day 0 | 276.1  236.1 | 287.0  245.4 | 297.4  254.4 | 333.0  289.6 | 345.7  300.2 | 307.8  265.1 | 30.1  28.2 | 0.04802 |
|  |  | 0819 Day 14  Day 14 – Day 0 | 366.9  326.9 | 394.0  352.4 | 432.8  389.8 | 454.9  411.5 | 462.1  416.6 | 422.1  379.4 | 40.7  38.8 | 0.03702 |
|  |  | 0821 Day 16  Day 16 – Day 0 | 462.5  422.5 | 521.6  480.0 | 543.2  500.2 | 564.2  520.8 | 586.7  541.2 | 535.6  492.9 | 47.5  45.5 | 0.02264 |
|  |  | 0824 Day 19  Day 19 – Day 0 | 589.7  549.7 | 624.8  583.2 | 717.9  674.9 | 714.6  671.2 | 773.9  728.4 | 684.2  641.5 | 75.1  73.1 | 0.04604 |
|  |  | 0826 Day 21  Day 21 – Day 0 | 773.6  733.6 | 826.6  785.0 | 979.5  936.5 | 974.1  930.7 | 1009.3  963.8 | 912.6  869.9 | 105.3  103.4 | 0.01978 |

| Group | Doses  (mg/kg) | Days | Animal number | | | | | Mean | Standard deviation | P-value  (t-TEST) |
| --- | --- | --- | --- | --- | --- | --- | --- | --- | --- | --- |
|  |  |  | 1 | 2 | 3 | 4 | 5 |  |  |  |
| pHLlP-miR-29a | 4 | 0805 Day 0  Day 0 – Day 0 | 40.0  0.0 | 41.6  0.0 | 42.6  0.0 | 43.3  0.0 | 46.1  0.0 | 42.7  0.0 | 2.3  0.0 |  |
|  |  | 0807 Day 2  Day 2 – Day 0 | 59.1  19.1 | 61.4  19.8 | 60.5  17.9 | 58.8  15.5 | 62.4  16.3 | 60.4  17.7 | 1.5  1.8 | 0.02292 |
|  |  | 0810 Day 5  Day 5 – Day 0 | 88.1  48.1 | 90.0  48.4 | 87.7  45.1 | 92.3  49.0 | 94.2  48.1 | 90.5  47.7 | 2.8  1.5 | 0.01486 |
|  |  | 0812 Day 7  Day 7 – Day 0 | 128.7  88.7 | 118.2  76.6 | 112.3  69.7 | 131.0  87.7 | 137.1  91.0 | 125.5  82.7 | 10.0  9.2 | 0.00531 |
|  |  | 0814 Day 9  Day 9 – Day 0 | 163.4  123.4 | 158.9  117.3 | 155.5  112.9 | 170.0  126.7 | 181.8  135.7 | 165.9  123.2 | 10.4  8.8 | 0.00068 |
|  |  | 0817 Day 12  Day 12 – Day 0 | 267.7  227.7 | 260.3  218.7 | 236.0  193.4 | 299.2  255.9 | 307.1  261.0 | 274.1  231.3 | 29.2  27.8 | 0.00289 |
|  |  | 0819 Day 14  Day 14 – Day 0 | 359.1  319.1 | 387.9  346.3 | 304.3  261.7 | 377.0  333.7 | 394.2  348.1 | 364.5  321.8 | 36.2  35.5 | 0.00102 |
|  |  | 0821 Day 16  Day 16 – Day 0 | 432.3  392.3 | 479.5  437.9 | 382.9  340.3 | 471.4  428.1 | 467.2  421.1 | 446.7  403.9 | 39.9  39.4 | 0.00039 |
|  |  | 0824 Day 19  Day 19 – Day 0 | 494.5  454.5 | 568.5  526.9 | 535.3  492.7 | 608.4  565.1 | 542.3  496.2 | 549.8  507.1 | 42.2  41.4 | 0.00048 |
|  |  | 0826 Day 21  Day 21 – Day 0 | 591.2  551.2 | 706.9  665.3 | 713.5  670.9 | 787.4  744.1 | 631.6  585.5 | 686.1  643.4 | 76.5  76.2 | 0.00009 |
