# Supplementary material for: MicroRNA 29a therapy for CEACAM6-expressing lung adenocarcinoma
Source: BMC Cancer. 2023 Sep 8;23:843. doi: 10.1186/s12885-023-11352-w (PMC10492333; doi:10.1186/s12885-023-11352-w)
Supplement: Supplementary file 7 — Additional file 7. Supplementary Table 2. The tumor volume of each animal in Figure 4A. [file 12885_2023_11352_MOESM7_ESM.docx]

Supplementary Table 2. The tumor volume of each animal in Figure 4A

| Group | Dose  (mg/kg) | Days | Animal number | | | | | Mean | Standard deviation | P-value  (t-TEST) |
| --- | --- | --- | --- | --- | --- | --- | --- | --- | --- | --- |
|  |  |  | 1 | 2 | 3 | 4 | 5 |  |  |  |
| Vehicle | 0 | 0620 Day 0  Day 0-Day 0 | 43.1  0.0 | 45.5  0.0 | 45.8  0.0 | 47.9  0.0 | 47.9  0.0 | 46.0  0.0 | 2.0  0.0 |  |
|  |  | 0622 Day 2  Day 2-Day 0 | 75.6  32.5 | 76.7  31.2 | 79.8  34.0 | 76.4  28.5 | 87.2  39.3 | 79.1  33.1 | 4.8  4.0 |  |
|  |  | 0624 Day 4  Day 4-Day 0 | 116.6  73.5 | 122.3  76.8 | 133.8  88.0 | 122.1  74.2 | 133.6  85.7 | 125.7  79.6 | 7.7  6.7 |  |
|  |  | 0627 Day 7  Day 7-Day 0 | 204.4  161.3 | 220.0  174.5 | 241.5  195.7 | 215.7  167.8 | 234.1  186.2 | 223.1  177.1 | 14.8  13.9 |  |
|  |  | 0629 Day 9  Day 9-Day 0 | 337.6  294.5 | 358.4  312.9 | 333.9  288.1 | 302.2  254.3 | 324.9  277.0 | 331.4  285.4 | 20.4  21.7 |  |
|  |  | 0701 Day 11  Day 11-Day 0 | 511.2  468.1 | 558.0  512.5 | 514.4  468.6 | 463.0  415.1 | 537.3  489.4 | 516.8  470.7 | 35.5  36.1 |  |
|  |  | 0704 Day 14  Day 14-Day 0 | 723.1  680.0 | 766.7  721.2 | 914.5  868.7 | 732.5  684.6 | 784.5  736.6 | 784.3  738.2 | 77.0  76.8 |  |
|  |  | 0706 Day 16  Day 16-Day 0 | 915.6  872.5 | 1009.1  963.6 | 1133.0  1087.2 | 979.4  931.5 | 995.4  947.5 | 1006.5  960.5 | 79.3  78.8 |  |
|  |  | 0708 Day 18  Day 18-Day 0 | 1137.5  1094.4 | 1237.9  1192.4 | 1341.4  1295.6 | 1283.1  1235.2 | 1129.7  1081.8 | 1225.9  1179.9 | 92.0  91.6 |  |
|  |  | 0711 Day 21  Day 21-Day 0 | 1479.3  1436.2 | 1521.0  1475.5 | 1581.7  1535.9 | 1639.4  1591.5 | 1383.3  1335.4 | 1520.9  1474.9 | 98.0  97.8 |  |

| Group | Dose  (mg/kg) | Days | Animal number | | | | | Mean | Standard deviation | P-value  (t-TEST) |
| --- | --- | --- | --- | --- | --- | --- | --- | --- | --- | --- |
|  |  |  | 1 | 2 | 3 | 4 | 5 |  |  |  |
| miR-29a | 2 | 0620 Day 0  Day 0-Day 0 | 43.2  0.0 | 45.4  0.0 | 46.1  0.0 | 47.3  0.0 | 48.8  0.0 | 46.2  0.0 | 2.1  0.0 |  |
|  |  | 0622 Day 2  Day 2-Day 0 | 77.9  34.7 | 72.2  26.8 | 75.6  29.5 | 79.0  31.7 | 81.5  32.7 | 77.2  31.1 | 3.5  3.0 | 0.39565 |
|  |  | 0624 Day 4  Day 4-Day 0 | 120.7  77.5 | 113.0  67.6 | 113.2  67.1 | 116.3  69.0 | 122.6  73.8 | 117.2  71.0 | 4.4  4.5 | 0.04431 |
|  |  | 0627 Day 7  Day 7-Day 0 | 206.4  163.2 | 181.4  136.0 | 189.9  143.8 | 218.1  170.8 | 198.1  149.3 | 198.8  152.6 | 14.3  14.2 | 0.02487 |
|  |  | 0629 Day 9  Day 9-Day 0 | 316.0  272.8 | 269.4  224.0 | 274.1  228.0 | 307.2  259.9 | 285.1  236.3 | 290.4  244.2 | 20.4  21.2 | 0.01622 |
|  |  | 0701 Day 11  Day 11-Day 0 | 488.6  445.4 | 395.5  350.1 | 435.2  389.1 | 457.4  410.1 | 447.1  398.3 | 444.8  398.6 | 33.9  34.5 | 0.01202 |
|  |  | 0704 Day 14  Day 14-Day 0 | 733.1  689.9 | 553.7  508.3 | 640.5  594.4 | 688.6  641.3 | 716.2  667.4 | 666.4  620.3 | 72.1  72.0 | 0.03658 |
|  |  | 0706 Day 16  Day 16-Day 0 | 866.1  822.9 | 790.1  744.7 | 828.7  782.6 | 853.8  806.5 | 882.5  833.7 | 844.2  798.1 | 36.1  35.5 | 0.00299 |
|  |  | 0708 Day 18  Day 18-Day 0 | 1060.0  1016.8 | 949.2  903.8 | 1017.5  971.4 | 1031.4  984.1 | 1064.0  1015.2 | 1024.4  978.3 | 46.3  46.0 | 0.00229 |
|  |  | 0711 Day 21  Day 21-Day 0 | 1307.3  1264.1 | 1234.8  1189.4 | 1164.3  1118.2 | 1331.8  1284.5 | 1266.5  1217.7 | 1260.9  1214.8 | 65.6  65.7 | 0.00114 |

| Group | Dose  (mg/kg) | Days | Animal number | | | | | Mean | Standard deviation | P-value  (t-TEST) |
| --- | --- | --- | --- | --- | --- | --- | --- | --- | --- | --- |
|  |  |  | 1 | 2 | 3 | 4 | 5 |  |  |  |
| Cisplatin | 2 | 0620 Day 0  Day 0-Day 0 | 42.4  0.0 | 43.7  0.0 | 47.2  0.0 | 47.2  0.0 | 49.8  0.0 | 46.1  0.0 | 3.0  0.0 |  |
|  |  | 0622 Day 2  Day 2-Day 0 | 72.3  29.9 | 80.3  36.6 | 72.9  25.7 | 76.6  29.4 | 78.9  29.1 | 76.2  30.1 | 3.5  4.0 | 0.27481 |
|  |  | 0624 Day 4  Day 4-Day 0 | 114.6  72.2 | 111.5  67.8 | 110.1  62.9 | 119.1  71.9 | 120.7  70.9 | 115.2  69.1 | 4.6  3.9 | 0.01672 |
|  |  | 0627 Day 7  Day 7-Day 0 | 204.3  161.9 | 208.8  165.1 | 177.4  130.2 | 198.1  150.9 | 187.2  137.4 | 195.2  149.1 | 12.8  15.1 | 0.01589 |
|  |  | 0629 Day 9  Day 9-Day 0 | 301.3  258.9 | 313.2  269.5 | 249.1  201.9 | 282.5  235.3 | 265.7  215.9 | 282.4  236.3 | 26.0  28.3 | 0.01528 |
|  |  | 0701 Day 11  Day 11-Day 0 | 433.7  391.3 | 481.4  437.7 | 374.0  326.8 | 431.1  383.9 | 418.2  368.4 | 427.7  381.6 | 38.4  40.1 | 0.00607 |
|  |  | 0704 Day 14  Day 14-Day 0 | 640.5  598.1 | 727.0  683.3 | 551.8  504.6 | 602.9  555.7 | 664.8  615.0 | 637.4  591.3 | 65.8  66.8 | 0.01211 |
|  |  | 0706 Day 16  Day 16-Day 0 | 796.8  754.4 | 862.7  819.0 | 749.7  702.5 | 770.6  723.4 | 814.3  764.5 | 798.8  752.8 | 43.4  44.5 | 0.00089 |
|  |  | 0708 Day 18  Day 18-Day 0 | 965.9  923.5 | 1024.1  980.4 | 882.9  835.7 | 899.1  851.9 | 934.4  884.6 | 941.3  895.2 | 56.3  58.3 | 0.00038 |
|  |  | 0711 Day 21  Day 21-Day 0 | 1145.5  1103.1 | 1207.0  1163.3 | 1095.0  1047.8 | 1034.9  987.7 | 1152.5  1102.7 | 1127.0  1080.9 | 65.0  66.2 | 0.00007 |

| Group | Dose  (mg/kg) | Days | Animal number | | | | | Mean | Standard deviation | P-value  (t-TEST) |
| --- | --- | --- | --- | --- | --- | --- | --- | --- | --- | --- |
|  |  |  | 1 | 2 | 3 | 4 | 5 |  |  |  |
| miR-29a  + Cisplatin | 2  + 2 | 0620 Day 0  Day 0-Day 0 | 42.9  0.0 | 44.9  0.0 | 46.3  0.0 | 47.3  0.0 | 48.9  0.0 | 46.1  0.0 | 2.3  0.0 |  |
|  |  | 0622 Day 2  Day 2-Day 0 | 71.0  28.1 | 73.1  28.2 | 76.6  30.3 | 79.0  31.7 | 74.4  25.5 | 74.8  28.8 | 3.1  2.4 | 0.07072 |
|  |  | 0624 Day 4  Day 4-Day 0 | 104.6  61.7 | 110.4  65.5 | 107.6  61.3 | 113.2  65.9 | 109.1  60.2 | 109.0  62.9 | 3.2  2.6 | 0.00085 |
|  |  | 0627 Day 7  Day 7-Day 0 | 169.5  126.6 | 187.2  142.3 | 160.2  113.9 | 193.5  146.2 | 191.6  142.7 | 180.4  134.3 | 14.7  13.7 | 0.00119 |
|  |  | 0629 Day 9  Day 9-Day 0 | 229.6  186.7 | 250.8  205.9 | 222.4  176.1 | 272.2  224.9 | 266.8  217.9 | 248.4  202.3 | 22.0  20.6 | 0.00026 |
|  |  | 0701 Day 11  Day 11-Day 0 | 338.6  295.7 | 369.5  324.6 | 332.6  286.3 | 394.3  347.0 | 422.6  373.7 | 371.5  325.5 | 37.9  36.1 | 0.00022 |
|  |  | 0704 Day 14  Day 14-Day 0 | 552.5  509.6 | 578.6  533.7 | 495.0  448.7 | 562.8  515.5 | 515.6  466.7 | 540.9  494.8 | 34.6  35.6 | 0.00020 |
|  |  | 0706 Day 16  Day 16-Day 0 | 672.3  629.4 | 699.3  654.4 | 581.9  535.6 | 723.2  675.9 | 686.3  637.4 | 672.6  626.5 | 54.1  53.9 | 0.00005 |
|  |  | 0708 Day 18  Day 18-Day 0 | 802.4  759.5 | 818.0  773.1 | 716.7  670.4 | 854.4  807.1 | 782.1  733.2 | 794.7  748.7 | 51.0  51.2 | 0.00001 |
|  |  | 0711 Day 21  Day 21-Day 0 | 976.2  933.3 | 1024.0  979.1 | 817.9  771.6 | 958.8  911.5 | 1013.5  964.6 | 958.1  912.0 | 82.8  82.8 | 0.00001 |
