# Supplementary material for: MicroRNA 29a therapy for CEACAM6-expressing lung adenocarcinoma
Source: BMC Cancer. 2023 Sep 8;23:843. doi: 10.1186/s12885-023-11352-w (PMC10492333; doi:10.1186/s12885-023-11352-w)

Figure 1A

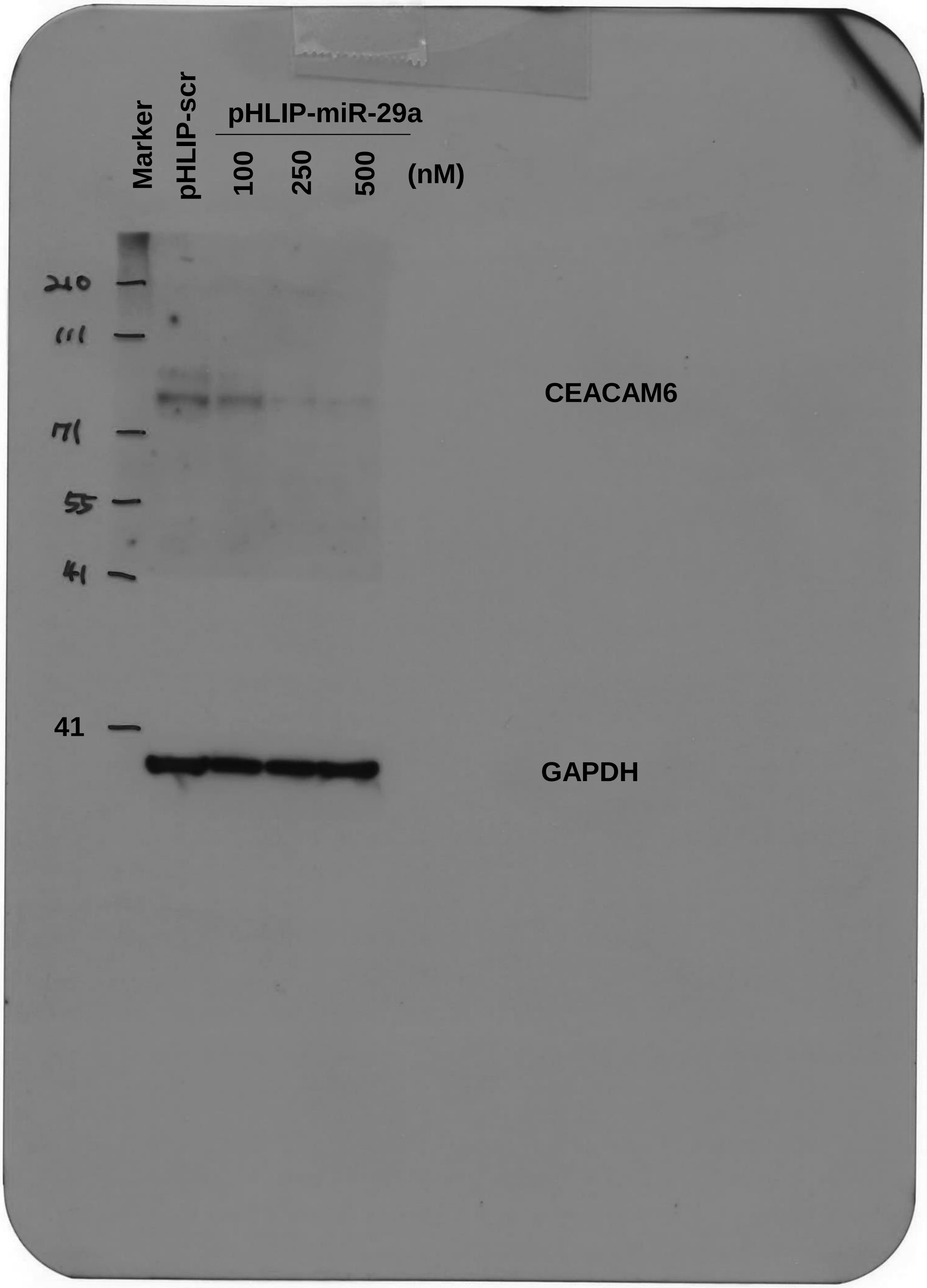

Marker
pHLIP-scr
pHLIP-miR-29a
100
250
500
(nM)
CEACAM6
41
GAPDH

Supplementary Figure 2A

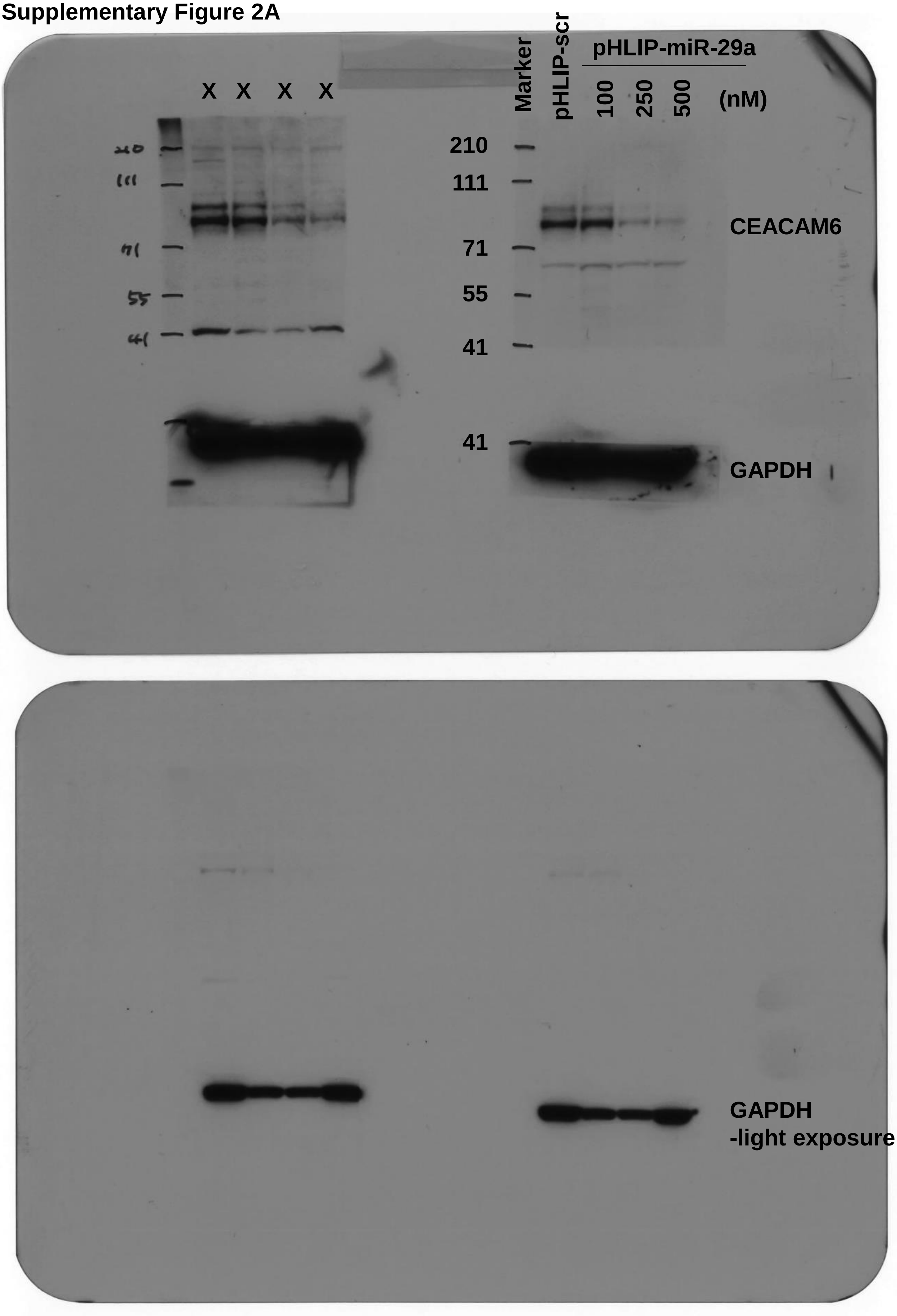

Supplementary Figure 3

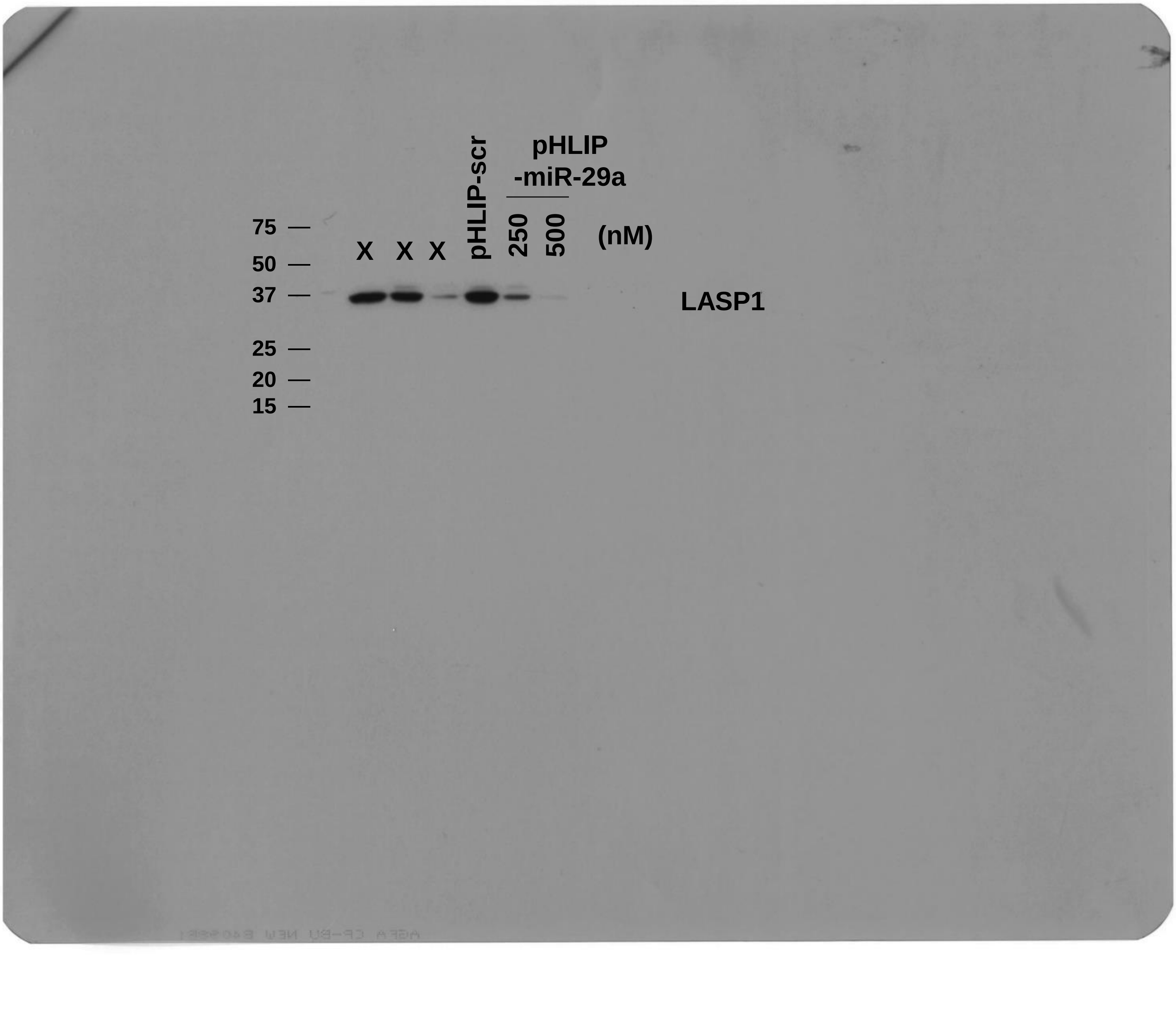

Supplementary Figure 3

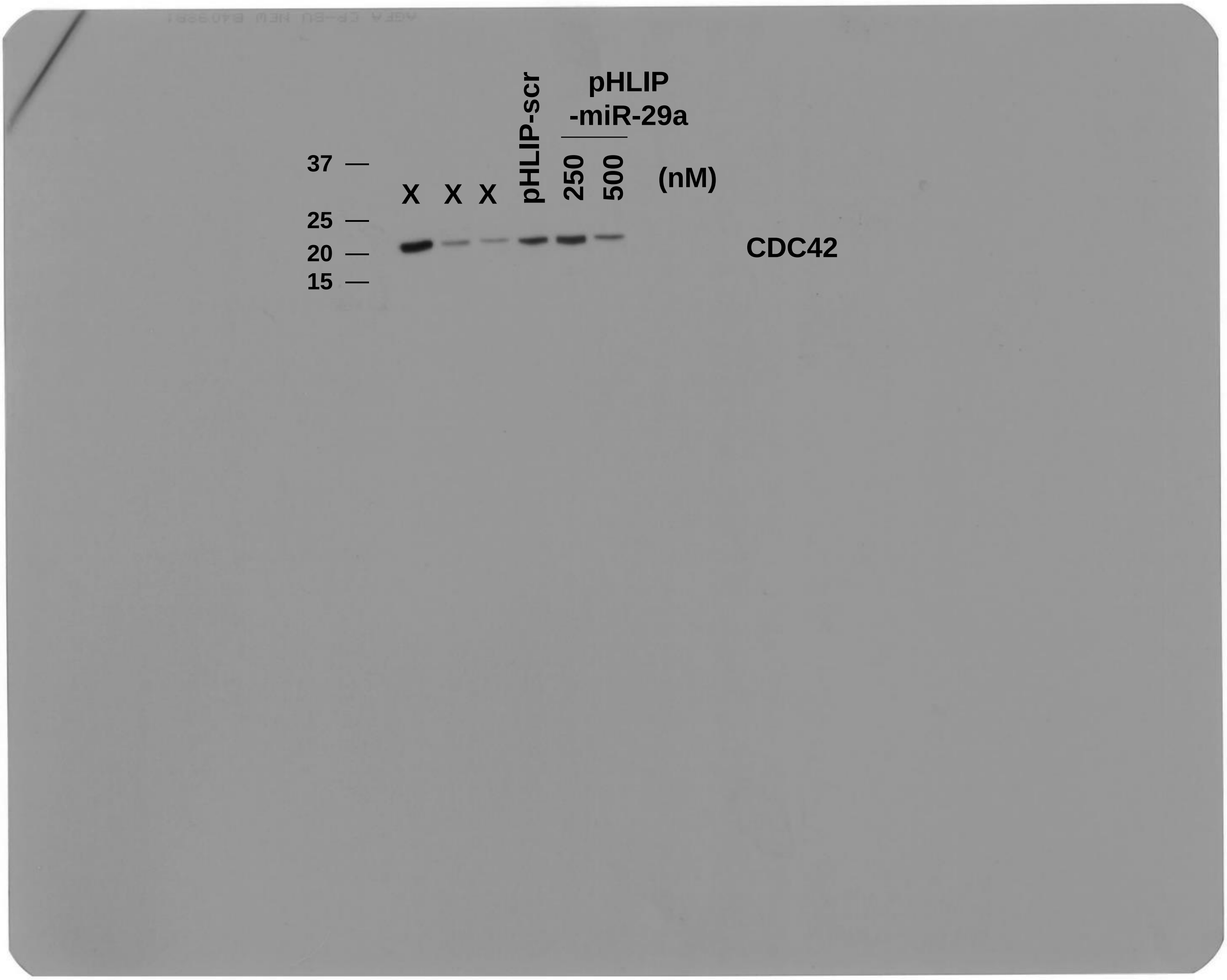

Supplementary Figure 3

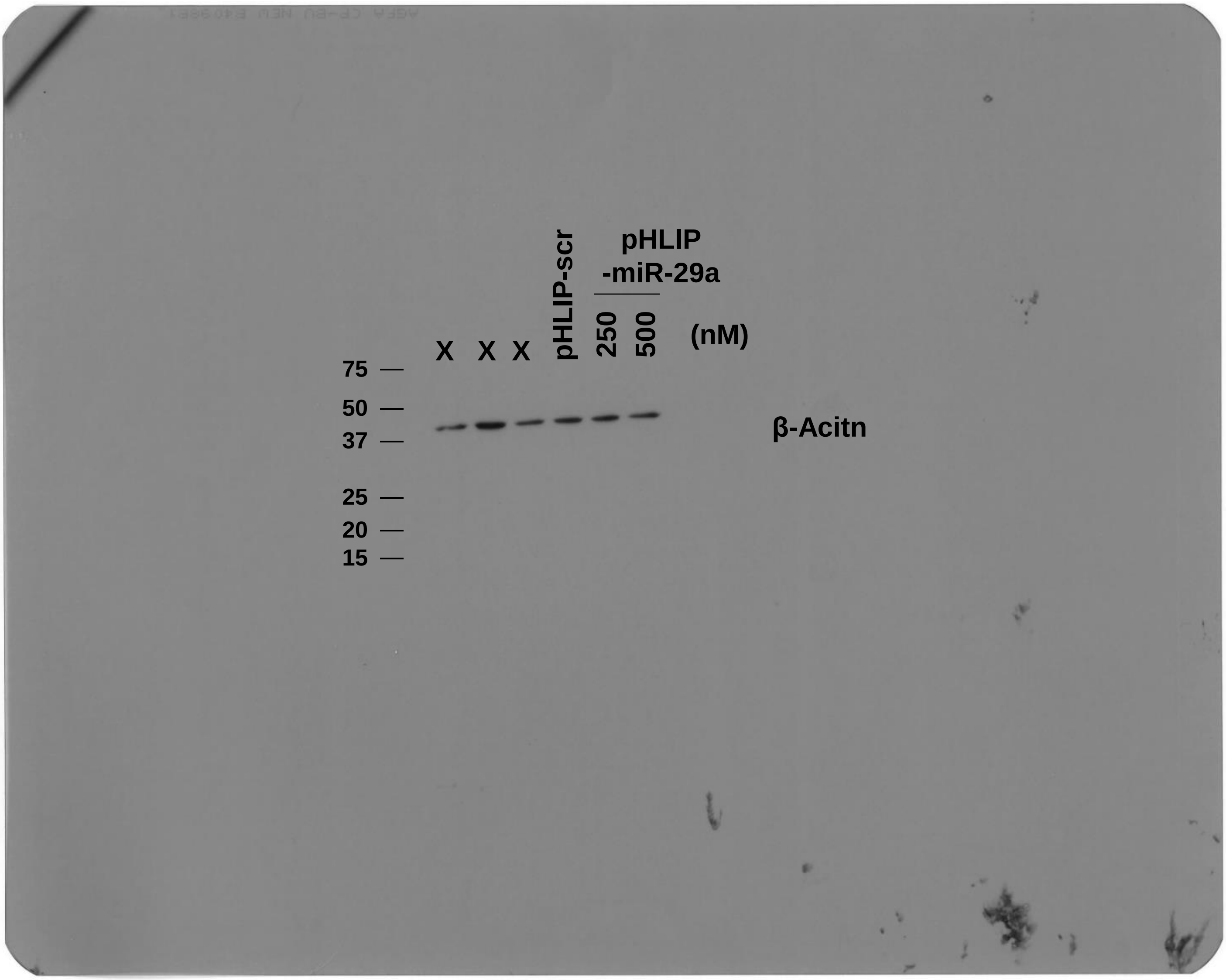

Supplement: Supplementary file 8 — Additional file 8. Raw images. [file 12885_2023_11352_MOESM8_ESM.pdf]
